# Superstructure formation by RodZ hexamers of *Shigella sonnei* maintains the rod shape of bacilli

**DOI:** 10.1371/journal.pone.0228052

**Published:** 2020-02-13

**Authors:** Jiro Mitobe, Fumiko Nishiumi, Itaru Yanagihara, Shouji Yamamoto, Makoto Ohnishi

**Affiliations:** 1 Department of Bacteriology I, National Institute of Infectious Diseases, Shinjuku, Tokyo, Japan; 2 Department of Developmental Medicine, Research Institute, Osaka Women’s and Children’s Hospital, Izumi, Osaka, Japan; University of Manchester, UNITED KINGDOM

## Abstract

The rod shape of bacilli is maintained by bacterial cytoskeletal protein MreB, an actin homolog that acts in concert with the inner membrane protein RodZ. We previously reported RodZ binds RNA to control the posttranscriptional regulation of *invE* (*virB*), which controls the type III secretion system essential for the virulence of *Shigella*. Here, we show that purified RodZ forms “superstructures” of high molecular mass that dissociate into a midsized “basal complex” in the presence of nonionic detergent, or to a monomer in the presence of dithiothreitol. We used mass spectrometry to show that the basal complex was a hexamer. Electrophoresis mobility shift assays combined with gel filtration detected the RNA-binding activity in fractions containing molecules larger than the basal hexamer. The superstructure was consistently detected with MreB in crude cell lysates of *S*. *sonnei* that were fractionated using gel filtration. Immunofluorescence microscopy using two different super-resolution settings showed that wild-type RodZ was distributed in cells as separate dots. Consistent with the superstructure comprising homohexamers, majority of the dots distributed among areas of discrete values. In addition, simultaneous immunodetection of MreB provided the first evidence of colocalization with RodZ as larger patch like signals. These findings indicate that native RodZ forms clusters of various sizes, which may correspond to a superstructure comprising multiple hexamers required for the RNA-binding activity.

## Introduction

The bacterial cytoskeletal protein MreB, an actin homolog, maintains the rod shape of bacilli [1–3]. The bacterial cytoskeleton comprises a set of proteins capable of polymerizing into a filamentous structure within cytosol to maintain cell shape and division [4, 5]. MreB mediates cell shape through interaction with the inner membrane protein RodZ [6–8], which was suggested by bacterial two hybrid assay [8] and isolation of suppressor mutation in *mreB* for *rodZ* deletion mutant of an *E*. *coli* K-12 strain [9]. RodZ tethers periplasmic factors for peptidoglycan synthesis, including penicillin binding proteins, essential for the synthesis of the cell wall [6–8].

Massive observation of cell shapes and the localization of MreB in *E*. *coli* indicate that MreB does not localized to cell poles, but preferentially localizes to inwardly curved regions and is excluded from bulging *vice versa*. This indicates that MreB localizes in response to local shape cues to maintain cylindrical uniformity [10]. In the absence of RodZ, MreB loses the curvature preference, and the cell lose its rod shape [11]. To adjust for changes in cell shape during proliferation, RodZ modulates the biophysical properties of MreB, resulting in changes of curvature-based localization in a growth-dependent manner [12].

*Shigella* species are difficult to genetically distinguish from *E*. *coli* [13] and the *rodZ* sequence is identical. We analyzed the virulence of *S*. *sonnei* harboring *rodZ* mutations, because deletion mutation of *rodZ* greatly affects the expression of the type III secretion system (T3SS), a major virulence factor essential for bacterial invasion into colonic epithelial cells to cause the bloody diarrhea of shigellosis. Expression of T3SS is regulated by temperature [14, 15] and osmolarity [16, 17] through posttranscriptional regulation of the virulence-factor activator *invE* (*virB*) by the RNA-binding protein Hfq.

RodZ was discovered in a Tn*5*-screening that recovers the expression of T3SS in a deletion mutant of c*pxA*, encoding a sensor of the bacterial two-component system. In this mutant, *invE* mRNA is transcribed without protein synthesis that results from enhanced production of RodZ in the Δ*cpxA* mutant [18, 19]. Hfq and RodZ similarly affect the regulation of the T3SS, because deletion of both *hfq* and *rodZ* recovers InvE production under the repressive conditions of the T3SS at low temperature [15] and low osmolarity [17]. Further, overexpression of Hfq and RodZ represses the synthesis of InvE when the T3SS is active [15, 19]. Consistent with these findings, purified RodZ (and Hfq) binds a synthetic RNA containing the *invE* sequence through a KRRKKR sequence in a region between the cytosolic and transmembrane domains of RodZ [19].

During its purification, we noticed that his_6_-tagged RodZ formed a soluble complex with a molecular mass higher than predicted (36.83 kDa). Consistent with this finding, other studies describe the biochemical and functional self-interactions of RodZ molecules [8, 9, 20]. However, we are unaware of systematic analyses of the mechanism of complex formation. To address this gap in our knowledge, here we used multiple methods to analyze complex formation by RodZ. We also used super-resolution immunofluorescence techniques to localize RodZ in bacterial cells and to determine its pattern of expression.

## Materials and methods

### Growth conditions

Luria-Bertani (LB) medium (LB Lenox; Difco #240230) was used to culture bacteria. ampicillin (100 μg/ ml, Wako #016–23301), kanamycin (25 μg/ ml, Wako #113–00343), and rifampicin (200 μg/ ml, Sigma-Aldrich #R3501), arabinose (25 μg/ ml, Wako #010–04582) was added as indicated. To prepare fresh cultures, a 1:200 dilution of an overnight culture that had been incubated at 30°C with shaking at 150 rpm, was inoculated into 5 ml of fresh media and harvested as indicated in the figures.

### Plasmid constructions

Primers were synthesized by Eurofins Genomics Japan. The DNA sequence of *rodZ* was amplified from MS390 (identical to nucleotides 2746386–2745373 of Genbank/EBI Data Bank accession number CP000038.1) using PCR with the primers listed in as follows: yfgA5(NdeI) 5'-GGAATTCCATATGAATACTGAAGCCACGCACG-3' and yfgA6(XhoI) 5'-CCGCTCGAGCTGCGCCGGTGATTGTTCGGC-3' for pET-rodZ (wild-type); yfgA5(NcoI) 5'-CATGCCATGGATACTGAAGCCACGCACG-3' and yfgAC84(XhoI) 5'-CCGCTCGAGCTTTTCCAGCCCTGGCAGCAGTTC-3' for pET-rodZ(RodZ_1–84_); yfgA5(NdeI) and yfgAC142(XhoI) 5'-CCGCTCGAGCTCTTCCTGCTGAGCTTTGTGGTCTTG-3' for pET-rodZ(RodZ_1–142_); yfgAN50(NdeI) and yfgAC155(XhoI) 5'-CCGCTCGAGGCTCAGTTCTGCCGAAGATTGATCG-3' for pET-rodZ(RodZ_50–155_); yfgAN50(NdeI) 5'-GGAATTCCATATGGATAAGGCACCCGCCGATCTTGCTTC-3' and yfgA6(XhoI) for pET-rodZ(RodZ_50–337_). Amplicons were cloned into pET22b (Novagen, Madison, WI, USA) to introduce a his_6_-tag at the C-terminal end. Substitutions for cysteine were generated from pBAD-rodZ _wt_ [19] using site-directed mutagenesis with the primer sets C38S-F 5'- GACTTTcCCTGAAGGTTTCCACGGTAC-3' and C38S-R, 5'- CTTCAGGgAAAGTCGCTCGGCAACGG-3' C263S-F 5'- GCCGATTcCTGGCTGGAGGTCACTGATG-3' and C263S-R 5'- CAGCCAGgAATCGGCAGTAAAGTTCATCAC-3'. The bacterial strains and plasmids are listed in Table 1.

**Table 1:** Bacterial strains and plasmids.

Bacterial strains	Genotype	Reference
MS390	*S*. *sonnei* wild-type strain	[18]
MS5201	MS390 Δ*rodZ*	[19]
MS5204	MS5201 (pBAD18-Kan)	[19]
MS5215	MS5201 (pBAD-rodZ)	[19]
MS5242	MS5201 (pBAD-rodZ_C38S_)	This study
MS5243	MS5201 (pBAD- rodZ_C263S_)	This study
MS5244	MS5201 (pBAD-rodZ_C38S, C263S_)	This study
**Plasmids**		
pBAD18-Kan		[21]
pBAD-rodZ_wt_	pBAD18-Kan carrying PCR-amplified *rodZ* of *S*. *sonnei*	[19]
pBAD-rodZ_C38S_	pBAD-rodZ carrying amino acid substitution C38S	This study
pBAD-rodZ_C263S_	pBAD-rodZ carrying amino acid substitution C263S	This study
pBAD-rodZ_C38S, C263S_	pBAD-rodZ carrying amino acids substitutions C38S, C263S	This study
pET22b		Novagen
pET-rodZ_wt_	pET22b carrying PCR-amplified *rodZ* of *S*.*sonnei*	[19]
pET-rodZ_1-84_	pET22b carrying the 1–84 amino acid region of *rodZ*	This study
pET-rodZ_1-142_	pET22b carrying the 1–142 amino acid region of *rodZ*	This study
pET-rodZ_50-155_	pET22b carrying the 50–155 amino acid region of *rodZ*	This study
pET-rodZ_50-337_	pET22b carrying the 50–337 amino acid region of *rodZ*	This study
pET-rodZ_ΔKRRKKR_	pET22b carrying *rodZ* with in-frame deletion of KRRKKR	[19]

### Protein purification

RodZ_wt_ was purified as previously described [19]. For large-scale preparations, this protocol was modified as follows: bacterial cells (BL21DE3 [Novagen] carrying pET-rodZ_wt_) from 500 mL culture in LB medium (0.5 mM IPTG for 2 hrs [19]) were suspended in 50 ml of 10 mM Tris HCl (pH 7.5), 0.2× cOmplete Protease Inhibitor Cocktail (Roche #11836170001), and passed twice through a French Press (20,000 psi, 4°C). The lysate was centrifuged at 3000 ×g for 3 min at 4°C to remove cell debris. Insoluble membrane fraction was collected by centrifugation at 20,000 ×g for 30 min at 4°C, then the pellet was dissolved in buffer A containing 0.1% Triton X-100 [19] and precipitated twice with (NH_4_)_2_SO_4_ to remove cellular RNA. The pellet was suspended in 15ml buffer B [19], desalted by HiPrep 26/10 desalting (GE #17508701) in a flow rate of 30 ml/min by Äkta Prime plus FPLC system. The protein fraction (ca. 30 ml, 15 mS>) was mixed with 10 ml P-11 phosphocellulose resin (Whatman, #4071–010, pretreated with 1M NaOH and HCl for 10 min, respectively), bind for 1 hr at 4°C with rotation, washed and eluted using 60 ml Buechner funnel (Iwaki #11G3). The eluted sample was applied to Protino Ni-TED 1000 affinity column (Macherey-Nagel, #745110.5) for concentration [19].

Deletion proteins of RodZ were commonly processed to the step of dissolving the protein samples in buffer B as previously described [19]. The samples expressed by the deletion proteins (RodZ_1–142_, RodZ_50–155_, and RodZ_50–337_) were desalted using the HiPrep desalting column and then loaded onto 5 ml P-11 resin prepacked in Econocolumn (Bio-Rad #7380015). Eluted protein was further purified using Protino Ni-TED 1000 chromatography. The deletion proteins RodZ_1–84_ and ΔKRRKKR were desalted and loaded onto a 5-ml HiTrap SP column (GE #17115101) and then fractionated using a buffer B containing 0.1 M–0.5 M NaCl. The eluted protein (0.3 M fraction) was dialyzed against buffer D (90 mM NaCl, 10 mM sodium phosphate, pH 7.4; 1% glycerol; and 0.1% Tween-20) overnight and then loaded onto a 5-ml Heparin Sepharose CL-6B (GE #17099801) in Econoclumn. Protein was eluted using buffer D containing 90 mM–300 mM NaCl. Eluted protein (150-mM NaCl fraction) was further purified using Protino Ni-TAD 1000. Protein concentrations were determined using Coomassie Brilliant Blue (CBB) staining after 5%–25% SDS-PAGE gels (S1 Fig), with bovine serum albumin (BSA) and lysozyme as standards.

### Protein analysis

Gel filtration of RodZ_wt_ and variants was performed using a SMART System (GE Healthcare) equipped with a Superdex 200 PC3.2/30 (GE #17108901) column under the following conditions: 16°C; flow rate, 40 μ1/min in RNA-binding buffer [19]. The Gel Filtration Standard (Bio-Rad #151–1901) was used. Electrophoresis mobility shift (EMS) analysis was performed as previously described [19]. For analytical ultracentrifugation, RodZ_wt_ (1 mg/ml) was centrifuged in a Beckman DU200 Ultracentrifuge at Katakura Industries Co., Ltd (Tokyo, Japan). The data were analyzed using the SDEFIT program [22]. Mutant proteins (5 μ1 each) were subjected to 3%–12% BN-PAGE using a NativePAGE Novex Bis-Tris Gel System (Invitrogen, USA) according to the manufacturer’s instructions. CovalX AG Laboratory Analytical Service (https://covalx.com/ Zurich Switzerland) determined the molecular mass of the complex. Ten serial dilutions (1 mg/ml–19.53 μg/ml) were prepared in 10 μl PBS, and an aliquot (1 μl) was analyzed using a Bruker Ultraflex III matrix-assisted laser desorption/ionization time-of-flight mass spectrometry (MALDI-TOF MS). For High-Mass MALDI MS, the sample (9 μl) was treated with K200 Stabilizer reagent (1 μl, 2 mg/ml) (CovalX K200 MALDI MS Analysis kit) and analyzed using the CovalX HM3 interaction module (calibrated with BSA and IgG), focusing on species ranging from 0 kDa to 1500 kDa. MS data were processed using CovalX’s Complex Tracker software (ver. 2.0), which corrects for the mass of the crosslinker. (See supporting information: CovalXforAMR2201212.pdf)

ITM Co (Japan) prepared the mouse monoclonal antibodies. Antibodies produced by clones 5–17 and 12–90 (mouse ascites) were suitable for immunoblotting and immunofluorescence microscopy, respectively. For immunoblotting, 5–17 was diluted 2000× in PBS containing 0.1% skim-milk processed as previously described [19]. 12–90 was diluted in 250× in Tris buffered saline (see later).

To prepare native RodZ, bacterial cells were suspended in 0.5 ml 1× RNA-binding buffer [19] containing 0.1% Tween 20, 0.1% Triton X-100, 0.5 mg/ml lysozyme and disrupted using an ultrasonic homogenizer (Branson 250D, USA). Micrococcal nuclease (80 units, Takara 2910A) was added to the lysate for 10 min at 4°C, after which the lysate was centrifuged at 20,000 ×g for 10 min, passed through a 0.45-μm filter, and then loaded (70 μl) onto the Superdex 200 PC3.2/30 using the RNA binding buffer containing 0.1% Tween 20 and 0.1% Triton X-100.

### Microscopy

Phase-contrast images were acquired using a BX-50 microscope (Olympus) with a UPlanFl 100× objective lens. Long and short axes of each cell (n ~ 300) were measured using the ImageJ Fiji (1.5.2), which calculates the average length and standard deviation and then generates a histogram (https://imagej.nih.gov/ij/docs/guide/146-2.html). The significance of differences between strains was analyzed using two tailed Student *t* test, with *p*<0.05 considered significant.

For immunofluorescence detection, the culture was fixed with 80% methanol as described [23] with modifications that improve the binding of bacterial cells as follows: PEI (1% solution, Sigma-Aldrich #P3143,) was added to coverslips (Matsunami, No. 1S HT, 0.17±0.005 mm) for 10 min, instead of adding poly-L-lysine to slide glasses. TBST (20 mM Tris HCl, pH 7.5, 150 mM NaCl, 0.05% Tween-20) was used for wash instead of PBST. Rabbit polyclonal antibody against MreB [9] was absorbed with methanol-fixed Δ*mreBCD* strain PA340-678 [24] as described previously [25]. The two primary antibodies were diluted 250 × in TBST containing 0.1% skim milk, incubated with bacterial cells on the coverslip for 1 hr at room temperature, and washed for 1 hr at 4°C.

For super resolution imaging using SIM, bacterial cells on the coverslip were incubated for 1 hr with secondary antibodies of 500 × Alexa Fluor 488-conjugated anti-mouse IgG (Thermo Fisher A28175) and Alexa Fluor 647-conjugated anti-rabbit IgG (Thermo Fisher A27040) at room temperature. The coverslips were mounted on a slide glass with a ProLong Diamond Antifade Mountant (Thermo Fisher), and images of multiple stacks (each 200 nm) were acquired using Spinning Disk Confocal Super Resolution Microscope Spin SR10 (Olympus) equipped with ORCA Flash4.0 V3 digital CMOS camera (Hamamatsu) and a UAPON100×OTIRF objective lens. Images were processed by ImageJ. All 5 stacks of each color channel were merged and presented as Fig 6A and 6B. Two color channels of the stack 1, 3 and 5 were merged and presented as Fig 6C. The areas (μm^2^) of each fluorescent signal was determined from total 230 cells of the wild-type strain. After calibration by “set scale” command entering the pixels length (124) for the micrograph scale bar (5 μm), images were opened in 600% magnification, selected the “Polygon Sections” tools to measure all fluorescent areas. Then, histogram for the distribution (μm^2^) were derived by “measurements” command from a list of the fluorescent areas, in which few areas larger than 0.1 μm^2^ were removed as artifact by merged signals.

For super resolution imaging using STORM, Alexa Fluor 647 conjugated anti mouse IgG (Abcam, #ab150115) was used for the secondary antibodies. The coverslip was soaked in chambers filled with quenching reagent, and images were collected using the N-STORM System (Nikon Instic, Inc.) equipped with an ORCA FLSH4.0 V2 digital CMOS camera (Hamamatsu) and SR apo TIRF 100×H objective lens.

## Results

### Formation of complexes by purified RodZ

**Fig 1:**
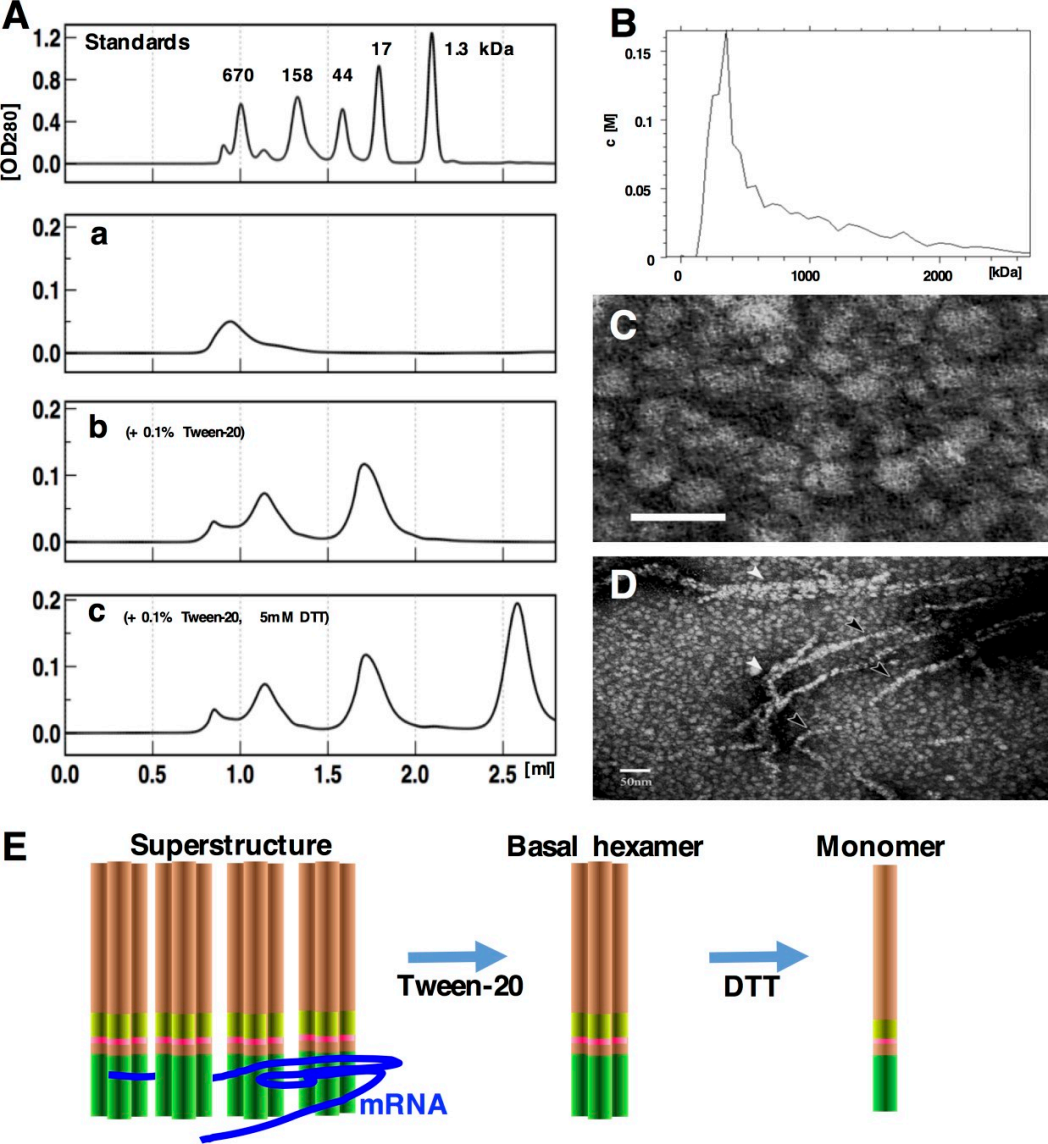
Multimer formation by RodZ. (A) Gel filtration of purified RodZ_wt:_ in RNA binding buffer [19]. Standards: Elution profile of molecular standards in the same condition: 670 kDa, thyroglobulin; 158 kDa, bovine γ-globulin; 44 kDa, ovalbumin; 17 kDa, myoglobin; 1.35 kDa, cyanocobalamin. a: Elution profile of RodZ_wt_ (0.5 mg/ml, 40 μl). b: Elution profile in RNA binding buffer containing 0.1% Tween-20. c: Elution profile in RNA binding buffer containing 0.1% Tween-20 and 5 mM dithiothreitol. (B) Analytical ultracentrifugation of RodZ_wt_ (1 mg/ml). (C) TEM image of purified RodZ_wt_. Scale bar = 25 nm. (D) Low magnification of the same sample. (E) Illustration for the three forms of RodZ complexes.

For characterization of the biochemical property of RodZ, an efficient purification procedure concentrating the membrane fraction was developed. During purification, we noticed RodZ was eluted as highly soluble complex, in which formation of insoluble aggregates was never detected at least concentrations ranging from 1 μg/ml to 1 mg/ml. The complex was examined by gel filtration analysis that detected a broad, major peak in fractions corresponding to the 1,000-kDa exclusion limit of the column (“large complex”) (Fig 1A, a).

RodZ possesses a transmembrane domain comprising hydrophobic amino acid residues 111–136 [6], suggesting that they participate in the associations between molecules. Consistent with this possibility, we detected a “midsized” complex that was released in the presence of a nonionic detergent (0.1% Tween-20: 16.7-times higher than critical micelle concentration (CMC_Tween20_) = 0.06 mg/ml [26]) between the 44-kDa and 17-kDa standards (Fig 1A, b).

**Fig 2:**
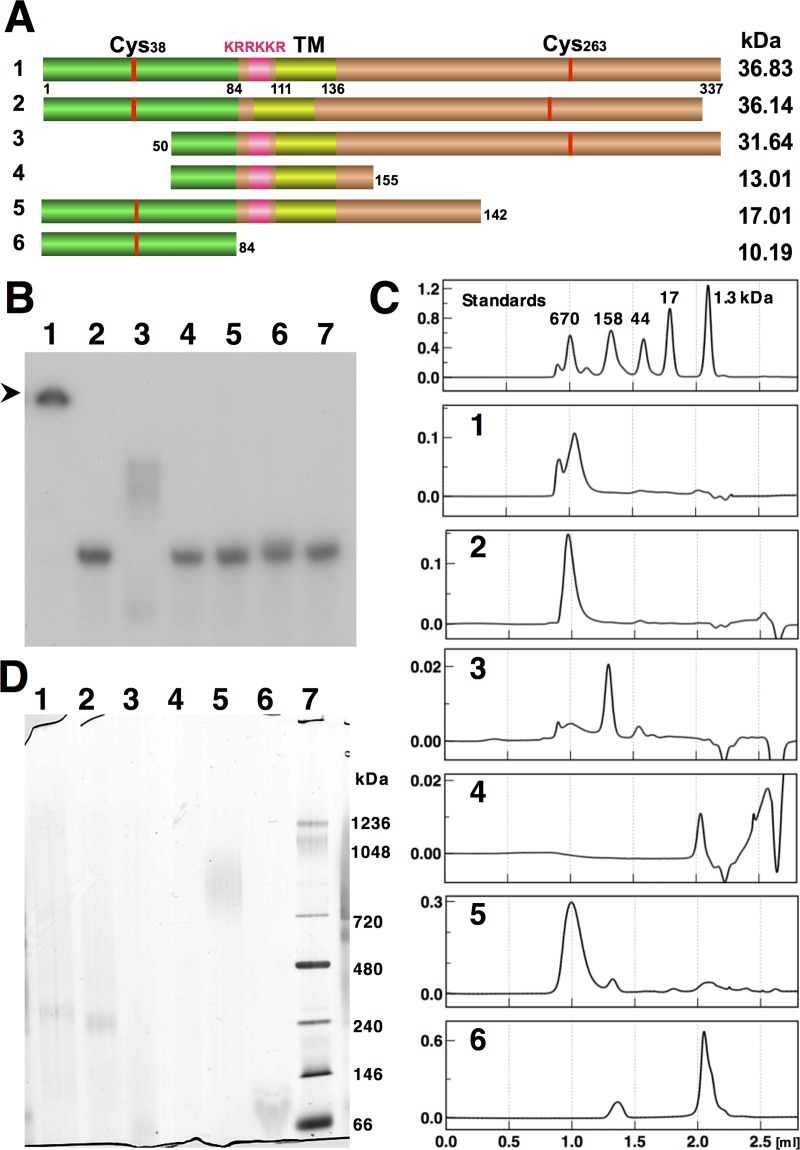
Functional mapping of the RNA-binding domain and multimerization of RodZ. The numbers indicating the RodZ variants are as follows: 1, RodZ_wt_; 2, RodZ_ΔKRRKKR_; 3, RodZ_50-337_; 4, RodZ_50-155_; 5, RodZ_1-142_; 6, RodZ_1-84_. (A) RodZ domains and deletion constructs: green, cytosolic; pink, KRRKKR; yellow, transmembrane; ocher, periplasmic domain; red, cysteine residues. Calculated molecular weights of the -his_6_ fusion proteins are indicated on the right side of the panel. (B) RNA binding activities of the indicated deletion proteins determined using EMS analysis. Lane 7: RNA probe without protein. Arrowhead indicates bottom of gell wells. (C) Gel filtration analysis of the deletion proteins. Standards: Elution profile of the molecular-weight standards is identical to that shown in Fig 1A. (D) BN-PAGE analysis of the deletion proteins. Lane 7: Molecular mass markers.

RodZ possesses two cysteine residues at positions 38 and 263 located in its cytosolic and periplasmic domains (Fig 2A), respectively [6]. Reducing condition in the cytosol generally prevent the formation of disulfide bonds [27]. Therefore, disulfide bonds may be formed between adjacent molecules through Cys_263_ at the periplasmic domain. Consistent with this possibility, we detected a new peak corresponding to a monomer in the presence of 5 mM dithiothreitol (Fig 1A, c). The peak of the monomer migrated slower than the smallest marker (1.3-kDa), and the midsized complex of putative multimer did not match the size of the complex likely because of nonspecific interactions with the column (Fig 1A, b and c).

These results indicate that RodZ formed three conformations (Fig 1E). Here we define the midsized complex as a basal complex and the large complex as a superstructure comprising several basal complexes. The superstructure was analyzed using ultracentrifugation (Fig 1B). Similar to the gel filtration results, ≥50% of the protein was distributed as a major peak ranging from 200 kDa to 600 kDa, decreasing in abundance toward 2,000 kDa.

When we subjected the same sample (75 μg/ml) to low-resolution transmission electron microscopy (TEM), we observed spherical particles with diameters ranging from 5 nm to 15 nm (Fig 1C), which likely correspond to the superstructure detected using analytical ultracentrifugation. We further observed filamentous structures ranging in thickness from 20–40 nm (Fig 1D, arrowheads). This fraction might correspond to the broadly distributed complexes >1,000 kDa detected using analytical ultracentrifugation (Fig 1B).

### Mutational analysis of RodZ complexes

To gain further information on the formation of RodZ multimers, we constructed plasmids encoding *rodZ* deletion proteins (Fig 2A) and purified the products (S1 Fig). Electrophoresis mobility shift (EMS) analysis was first performed to determine RNA-binding activity mediated by the sequence KRRKKR [19] (Fig 2B, lanes 1 and 2). Consistent with the formation of superstructure, the signal corresponding to the RNA-binding activity of RodZ was detected at the bottom of the gel-wells (Fig 2B, black arrowhead). This suggested the presence of high molecular mass species unable to enter the gel matrix even at the low concentration for EMS analysis (20 nM = 0.73 μg/ml). The deletion proteins RodZ_50–155_ (Fig 2B, lane 4) and RodZ_1–142_ (Fig 2B, lane 5) containing the KRRKKR sequence did not detectably bind the RNA probe. In contrast, the RodZ_50–337_ deletion protein (Fig 2B, lane 3) bound the RNA probe, indicated by the moderate shift of a few bands.

**Fig 3:**
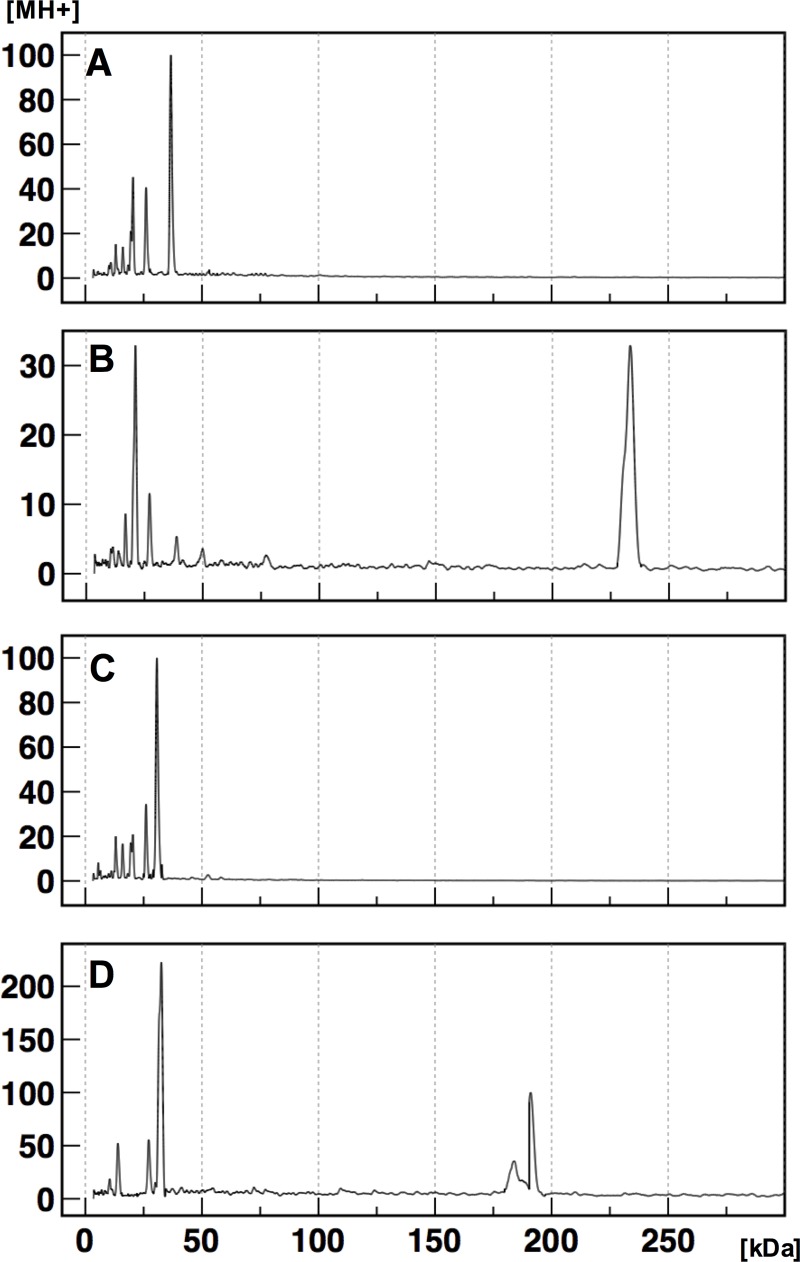
MALDI-TOF MS analysis of the high molecular mass complex. (A) Analysis without crosslinker of purified RodZ_wt_. (B) Analysis after treatment with crosslinker of purified RodZ_wt_. (C) Analysis without crosslinker of the purified N-terminal deletion RodZ_50-337_. (D) Analysis after treatment with crosslinker of the purified N-terminal deletion RodZ_50-337_. The data for 0–300 kDa species are presented here, and those for the entire mass range (0–1500 kDa) are shown in S2 Fig. Graphs show representative examples of serial 10-fold dilution of samples. The average values are stated in the Results section.

**Fig 4:**
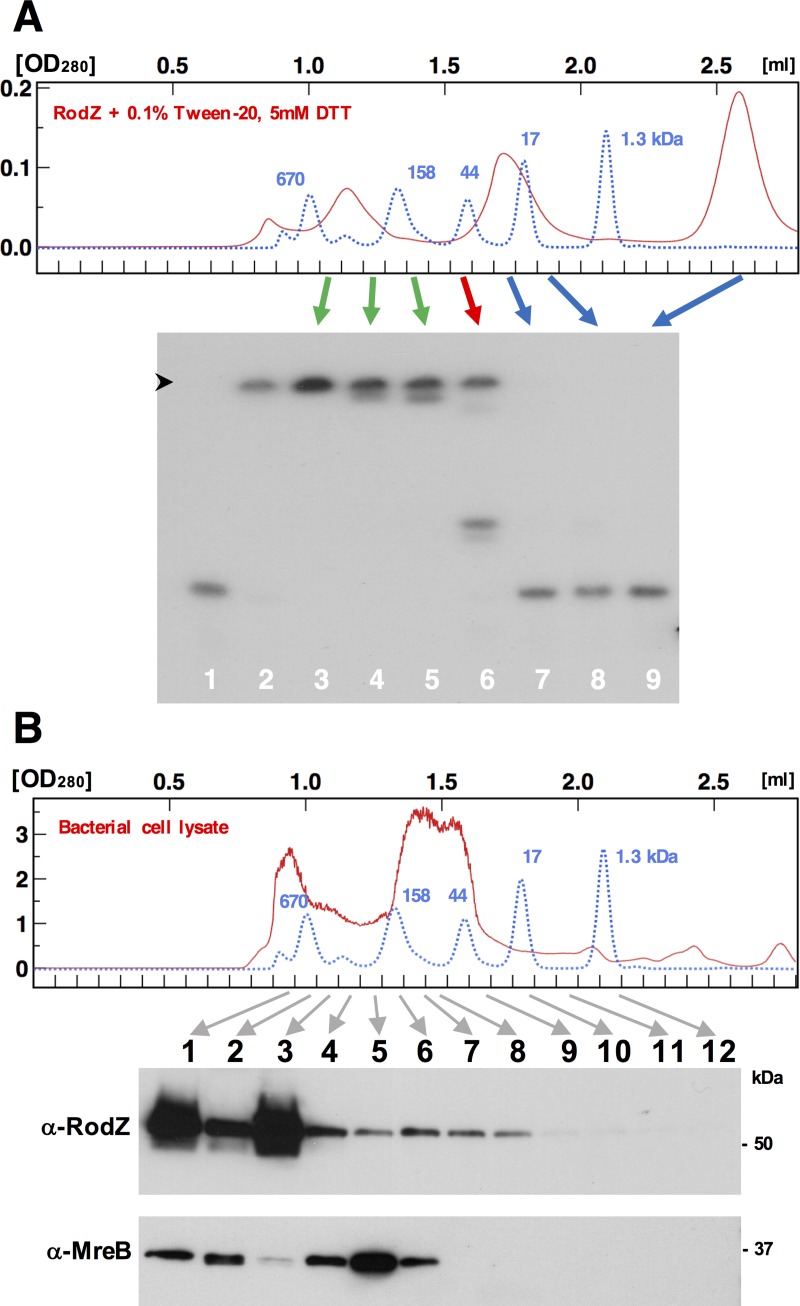
Multimer formation of RodZ *in vitro* and *in vivo*. (A) EMS analysis using fractions from the gel filtration column indicated in panel c of Fig 1A (Red line). The blue line indicates the superimposition of the molecular mass standards, identical to those in Fig 1A. Positions of the fractions used for EMS analysis are indicated by colored arrows. Lane 1: negative control without protein. Lane 2: positive control with unfractionated input. Black arrowhead indicates bottom of gel wells. (B) Soluble cell lysates were prepared by sonication and filtration of MS390 grown to midlogarithmic phase (OD_600_ = 0.5) in 35 ml LB medium at 37°C with shaking at 150 rpm (see Experimental procedures). Each 40-μl sample was fractionated using a Superdex-200 PC3.2/30 column equilibrated with the RNA binding buffer containing 0.1% Tween 20 and 0.1% Triton X-100 at flow rate of 40 μl/min (upper panel). The red line indicates the OD_280_ of the sample. The blue line indicates the superimposition of the molecular mass standards, identical to those in Fig 1A. Fractions indicated with arrows were subjected to immunoblotting using the anti-RodZ monoclonal antibody 5–17 and anti-MreB polyclonal antibody on the same blot of 10% SDS-PAGE (lower panels). Experiments were performed at least three times with similar results. Representative data are shown.

**Fig 5:**
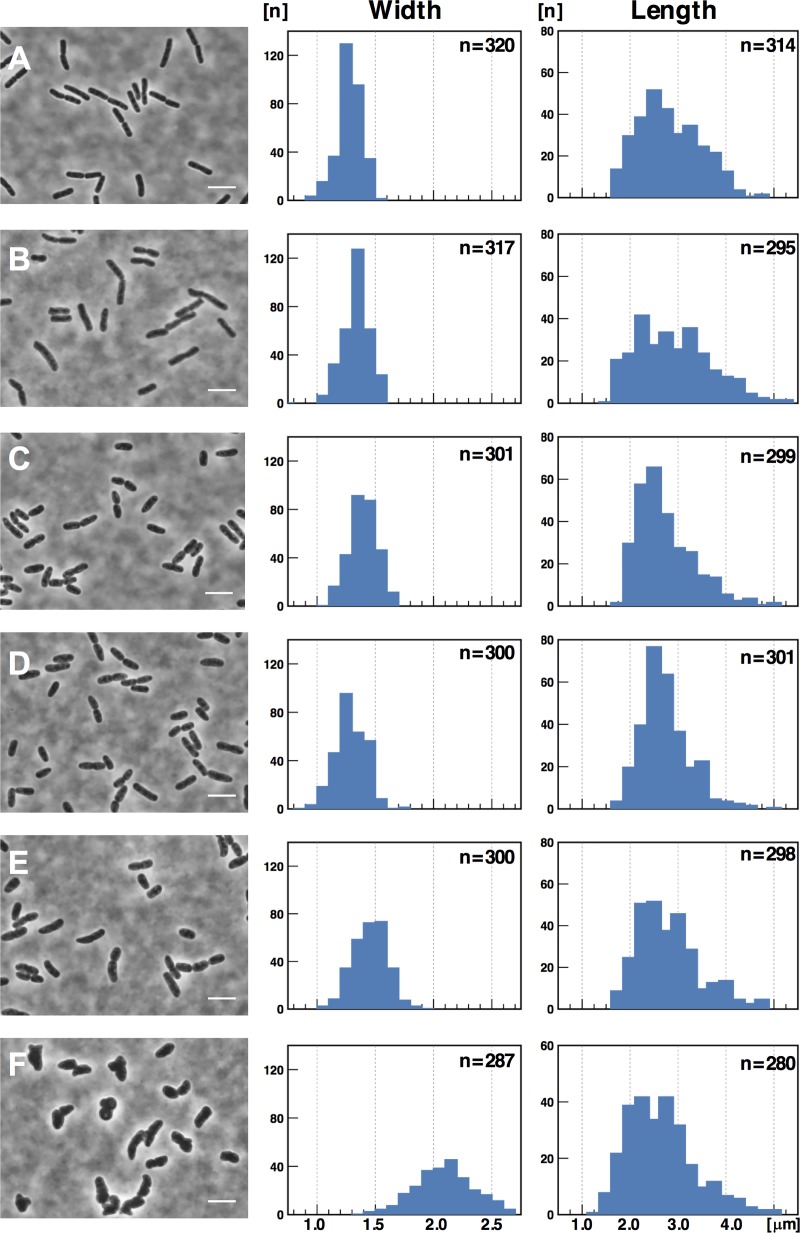
Shapes of the Δ*rodZ* mutant expressing RodZ cysteine-substitution mutants. (A) wild-type strain MS390. Δ*rodZ* strain carrying: (B) pBAD-rodZ_wt_ (MS5215); (C) pBAD-rodZ_C38S_ (MS5242); (D) pBAD-rodZ_C263S_ (MS5243); (E) pBAD-rodZ_C38S, C263S_ (MS5244); or (F) pBAD18-Kan (MS5204). Strains were grown to OD_600_ = 0.4 in 5-ml LB medium containing 25 μM arabinose and kanamycin at 30°C with shaking (150 rpm) for 2.5 hrs. Cells were fixed with 1.0% formaldehyde and observed using a phase-contrast microscope. Scale bar = 5 μm. The length distributions were analyzed using two tailed Student’s *t* test. Significance (p<0.05) differences were observed between all combinations of width and length, except for the combinations of lengths as follows: (C) and (D); (D) and (E); (C) and (E). Results are combination of three independent experiments.

**Fig 6:**
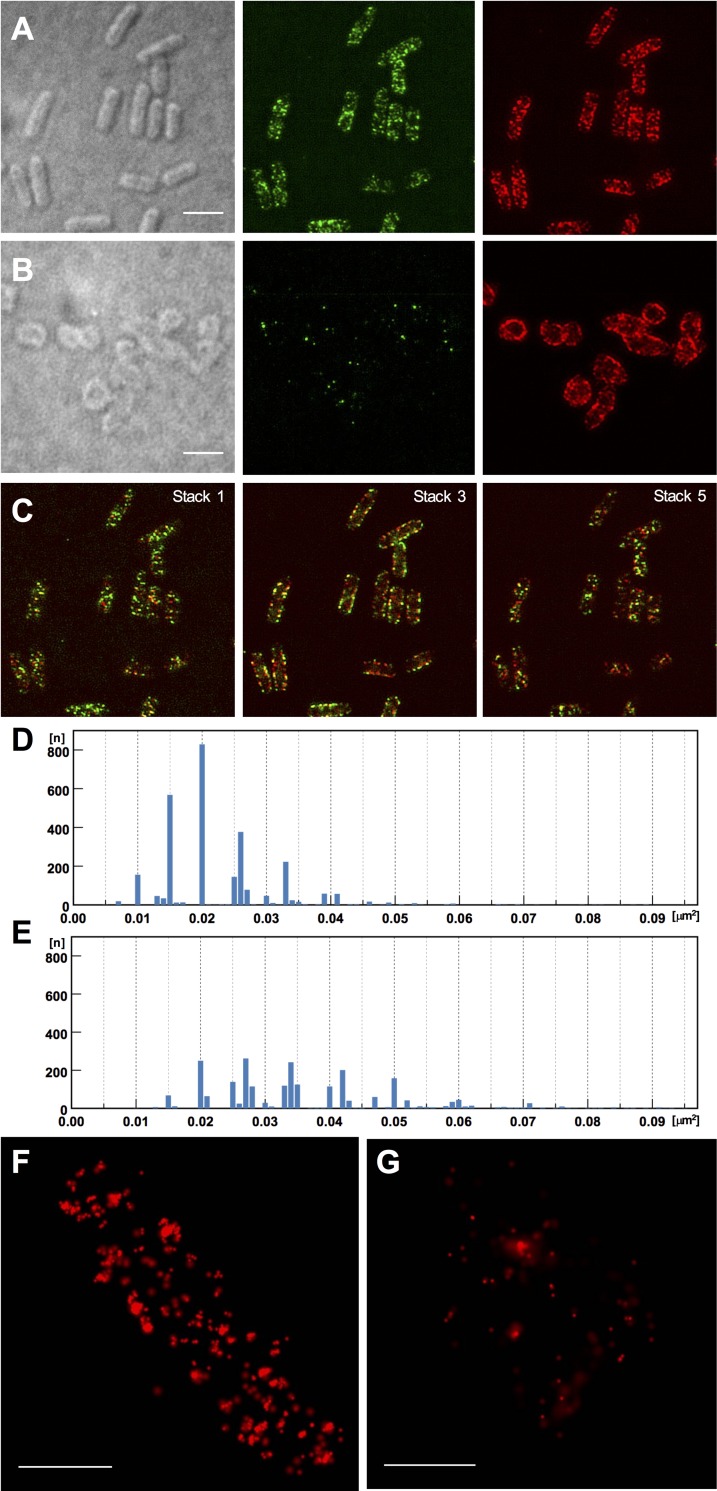
Super-resolution images of RodZ. Images acquired using SIM: Strains were grown to OD_600_ = 0.4 in 5-ml LB medium at 30°C with shaking (150 rpm) for 2.5 hrs. (A) wild-type strain MS390. (B) Δ*rodZ* strain MS5201. Panels: Left, differential interference contrast microscopy. Scale bar = 3 μm; Middle, Detection using the anti-RodZ 12–90 antibody and an anti-mouse Alexa Fluor 488 conjugate; Right, Detection using the anti-MreB polyclonal antibody and an anti-rabbit Alexa Fluor 647 conjugate. Signals of all 5 stacks (each 200 nm) were presented. (C) RodZ and MreB signals in stack 1, 3 and 5 of Fig 6A. (D) Distribution of 2811 dots from 230 wild-type cells observed using SIM (Mean = 0.023 μm^2^). (E) Distribution of 2358 patches observed using SIM (Mean = 0.036 μm^2^). Images were acquired using STORM: (F) MS390; (G) MS5201. Scale bar = 1 μm.

The deletion proteins were analyzed using gel filtration in the absence of Tween-20 and dithiothreitol. The RodZ_wt_ (Fig 2C, 1) and RodZ_ΔKRRKKR_ (Fig 2C, 2) eluted at similar positions. RodZ_50–337_ (Fig 2C, 3), which exhibited intermediate mobility in the EMS analysis, consistently eluted at a position similar to that of the 158-kDa marker, between the superstructure and the basal complex (Fig 1A, b). RodZ_50–155_ (Fig 2C, 4) eluted later, possibly because of its interactions with the column matrix. RodZ_1–142_ (Fig 2C, 5) eluted as a single peak at a position corresponding to the superstructure. RodZ_1–84_ (Fig 2C, 6) eluted as a large peak near the position of the 1.3-kDa marker as well as a small peak near the position of the 158-kDa marker.

The size of the complex was determined using Blue-native (BN) PAGE, in which CBB is included in the absence of SDS, and the loading buffer contains mild detergents [28]. Under these conditions, probable basal complexes of RodZ_wt_ and RodZ_ΔKRRKKR_ eluted near the 240-kDa marker, consistent with the expected size of a hexamer (Fig 2D, lanes 1 and 2). However, the background in the lanes made it difficult to arrive at definitive conclusions. RodZ_50–337_ (31.64 kDa) migrated near the 66-kDa standard (Fig 2D, lane 3) as possible dimer (See discussion). The second-smallest deletion protein RodZ_50–155_ was not detected (Fig 2D, lane 4). RodZ_1–142_ migrated as a broad band near the 1,000-kDa marker (Fig 2D, lane 5), and the smallest variant, RodZ_1–84_ (Fig 2D, lane 6), migrated between the 146-kDa and 66-kDa markers (Fig 2D, lane 6) as possible multimer.

### MALDI-TOF MS analysis

The combination of crosslinking with MALDI-TOF MS determines the molecular weights of complexes comprising proteins linked at the sites within the molecular distance that corresponds to disulfide bounds [29]. The crosslinked basal complex and monomers were detected by the dissociation of weak bonds during ionization. The average molecular mass of crosslinked RodZ_wt_ was 233.351 kDa (Fig 3B), corresponding to 6.36-times of the measurement of monomer (36.712 kDa) (Fig 3A). The broad distribution of crosslinked signals could be explained by excess binding to the crosslinker. Taking this into account, computer analysis determined that the true predicted molecular mass was 220.455 kDa, consistent with that of a hexamer (6.00-times).

RodZ_50-337_ (Fig 3D) was detected as peaks at 185.798 kDa and 191.059 kDa, equivalent to 6.05- and 6.22-times that of the monomer (30.692 kDa) (Fig 3C), respectively. The deduced molecular weight was 185.189 kDa (6.03-times).

### Function-structure analysis of multimer formation *in vitro*

To determine whether the hexamer bound RNA, the three forms of RodZ were fractionated using gel filtration (Fig 1A, c), and RNA-binding activity was determined using EMS analysis immediately after fractionation. The monomer fraction (Fig 4A, lane 9) failed to bind the RNA probe. Among the fractions corresponding to the basal complex (1.5–2.0 ml), only the fraction eluting at approximately 1.5 ml bound the RNA probe. Approximately 50% of the signal was detected in the middle of the EMS gel, suggesting that this fraction formed a low molecular mass complex that entered the gel. The failure of the top fractions containing the basal complex to bind RNA (Fig 4A, lanes 7 and 8) indicated that the basal hexamer itself did not bind RNA, thus suggesting that the association of multiple hexamers was required to bind RNA (Fig 4A, lanes 3, 4, and 5).

### Detection of the superstructure in a bacterial cell lysate

A crude extract of wild-type *S*. *sonnei* strain MS390 (referred to in the text that follows as MS390) was fractionated by gel filtration and detected RodZ complexes by immunoblotting [6]. RodZ consistently eluted near the 670-kDa marker corresponding to the superstructure (Fig 4B, blue line) but with two separate peaks. Although the buffer contained detergents, the peak corresponding to the basal complex was unclear. MreB in the same sample was fractionated among two separate peaks near the 670-kDa and 158-kDa markers, respectively. Detection of the larger peak suggested possible interaction with RodZ superstructure (see discussion). The smaller peaks including the main portion suggested multimar of MreB as described previously [12].

### Substitution of the two cysteine residues of RodZ affects cell shape

To determine the role of disulfide bonds in the structure and function of RodZ *in vivo*, the cysteine residues in pBAD-rodZ_wt_ [19] were substituted with serine residues. These substitutions were not affected the stability of the protein (S3 Fig). The single-substitution pBAD-rodZ_C38S_ and pBAD-rodZ_C263S_ constructs and the double-substitution pBAD-rodZ_C38S, C263S_ construct were introduced into *S*. *sonnei* strain MS5201 harboring a Δ*rodZ* deletion mutation. RodZ synthesis was induced by 25 μM arabinose, which produced amounts of RodZ similar to those of the wild-type strain as determined using immunodetection (S4 Fig). The shapes of the Δ*rodZ* mutants expressing these plasmids were observed using a phase-contrast microscope.

The distribution of cell widths shifted to a larger value in Δ*rodZ* cells (Fig 5F) as reported previously [20]. The width of cells transformed with each plasmid was significantly affected (p<0.05). The C38S (Fig 5C) substitution altered cell width more compared with that of the C263S substitution (Fig 5D), and the C38S and C263S had additive effects (p<0.05) (Fig 5E). Also, the cells length was significantly affected among the plasmid carrying strains (p<0.05) except following combinations: C38S and C263S (p = 0.72); C38S and C38, 263S (p = 0.30); C263S and C38, 263S (p = 0.14).

### Superstructure of the RodZ complex

We next determined the localization of RodZ in fixed bacterial cells. The size of the fluorescent signals were expected to be smaller than the diffraction limit of conventional light microscopy. Therefore, we used structured illumination microscopy (SIM), which achieves a resolution of 120 nm [30, 31] for detection of RodZ by anti-RodZ monoclonal antibody. Observations of the wild-type strain (Fig 6A) revealed that RodZ formed numerous dots of various sizes. Measurement of the signal areas exhibited that considerable number of dots distributed among areas with discrete values at 0.01, 0.015, 0.02, 0.026, and 0.033 μm^2^. (Fig 6D). The dots were more frequently distributed in diffused clusters than ordered helical-like strands. A few dots were detected in the Δ*rodZ* deletion strain MS5201, likely due to residual unreacted antibody (Fig 6B).

Bacterial cytoskeleton MreB was simultaneously observed using anti-MreB antibody [9] in the same wild-type cells. Signals of MreB were detected as larger “patches” at the same position to that of RodZ except for cell poles (Fig 6A). Distribution of MreB differed in Δ*rodZ* strain (Fig 6B) as reported previously [12], which manifested dense staining and loss of the patch-like distribution of signals. Observation of stack images (Fig 6C) indicated colocalization of both proteins, where considerable numbers of larger MreB patches and some RodZ dots were separately detected. Measurement of the areas indicated that the size distributed to larger extent than that of RodZ and clustering into specific values was not evident (Fig 6E).

Next, the intracellular distribution of RodZ was further examined using another setting of super-resolution by stochastic optical resolution microscopy (STORM), which enables further resolution (XY 20 nm, Z 50 nm) [32]. High-magnification observations of wild-type cells detected RodZ fluorescence as separated signals. The number of signals ranged from 200 to 300 per cell, indicating that numerous intracellular molecules were detected as individual dots (Fig 6F).

## Discussion

Most studies of RodZ focus on the function of cell shape formation and have provided abundant clues for resolving unanswered questions about the formation of bacterial shapes. Here we addressed the biophysical properties of RodZ. To produce sufficient amounts of RodZ, the purification scheme was first improved by concentrating the membrane fraction, which greatly simplified the purification procedure (see Experimental procedures).

Here we show the following: 1. RodZ formed a large complex (>200 kDa) that we call the superstructure (Fig 1E), which was detected using analytical ultracentrifugation, TEM, BN-PAGE, and MALDI-TOF MS. Gel filtration analysis of bacterial cell lysates showed that the large complex was similar to the superstructure detected in living cells. Consistent findings were acquired using super-resolution immunofluorescence microscopy, which detected corresponding signals in the form of scattered dots of various sizes. 2. The superstructure was formed by a two-step process involving hydrophobic interaction between basal hexamers and disulfide-bond formation among monomers that consistently affected the shapes of bacterial cells expressing substituted cysteine residues. 3. A complex of multiple hexamers was essential for RNA-binding activity *in vitro*. In contrast, hexamers alone did not bind RNA. Our future studies will focus on the association of the RNA-binding activity of RodZ with gene regulation as found for the RNA-binding protein Hfq that regulates the expression of *invE* (J. Mitobe, unpublished data).

We encountered a technical complication that may be explained by the properties of RodZ and its complexes as well as by the intrinsic difficulties associated with the analysis of membrane proteins. The same sample was used for gel filtration analyses, the weakest signals were detected in an experiment without detergent (Fig 1A, a), and additional peaks were detected in the presence of detergent and dithiothreitol. Further, the peaks eluted slower than the molecular size standards, suggesting that the elution of RodZ from the gel filtration columns was atypical. Although we did not encounter clogging of the column that would increase back pressure or decrease the flow rate, a considerable amount of bound protein was eluted by the SDS wash. These findings suggest incomplete recovery of the input protein, which may be accounted for nonspecific binding to the column under our elution conditions. Similar results were obtained using lower input protein concentrations. Therefore, we conclude that such a property was specific for our RodZ preparations.

The both substitutions C38S and C263S additively affected cell shape without affecting its protein stability Therefore, the altered cell shape might result from possible change of RodZ function. Our preliminary experiment suggested that purified RodZ without two cysteine resides appeared to keep the superstructure. If the RodZ form a possible superstructure without disulfide bonds, changes of cell shape may result from loss of unidentified function concerning the cysteine residues or loss of the chemical tightness of the hexamer. Further analysis will resolve the question. Cys_263_ is conserved among the RodZ molecules expressed by gammaproteobacteria, whereas Cys_38_ is present in RodZ molecules expressed in enterobacteriaceae such as *E*. *coli*, *Shigella*, *Salmonella*, *Yersinia*, and *Citrobacter* [7] suggesting the possibility of adaptation to a specific environment.

We show here that RodZ exists as three distinct forms. Specifically, ultracentrifugation studies detected a broad peak encompassing molecules ranging from 200 kDa to 500 kDa; BN-PAGE analysis detected a complex of approximately 240 kDa; and MALDI-TOF MS precisely identified the middle complex as a hexamer.

Analysis of deletion constructs provided clues to the assemble mechanism of the hexamers into the superstructure. In BN-PAGE analyses, RodZ_50–155_ was eluted from the gel, and possible multimer of RodZ_1–84_ migrated near the position of the 66-kDa marker, which was larger than the calculated size of 10.19 kDa (Fig 2A). These results indicate that the N-terminal domain initiates assemble of the basal complex. From unknown reason, RodZ_1–142_ was detected as the largest complex, suggesting the both N-terminal and transmembrane domains cooperate in assembly of a tight superstructure, which was resistant to the dissociative condition of BN-PAGE that released the basal complex for both wild-type and ΔKRRKKR constructs. RodZ_50–337_, which was crosslinked as hexamer (Fig 3D) but have single cysteine residue, migrated near the 66-kDa marker as a possible dimer probably due to the dissociative condition.

The complex formed by RodZ_50–337_ may comprise a limited number of hexamers, because it migrated at a position between the superstructure and the basal hexamer in gel filtration (Fig 2C-3), again suggesting the role of the N-terminal domain in the formation of the superstructure. The inability of RodZ_50–337_ to form the superstructure is also supported by the moderate shift detected by the EMS analysis (Fig 2B, lane 3), suggesting the presence of a “minimal RNA-binding unit” similar to the fraction coeluting at 1.5 ml in gel filtration analysis (Fig 4A, lane 6).

We previously found that the KRRKKR domain is essential for the RNA-binding activity of RodZ [19]. The analyses of deletion constructs here show that the C-terminal domains is indispensable for RNA-binding activity in addition to the KRRKKR domain. The variants without the C-terminal domain, but with the KRRKKR domain, failed to bind the RNA probe, suggesting that proper formation of the hexamer through the C-terminal domains of RodZ may bundle the KRRKKR domains, concentrating sufficient electrostatic charge for RNA-binding. Moreover, the association of several hexamers likely concentrates the charged residues to provide a niche suitable for binding RNAs.

The two-step assembly of RodZ, which was essential for RNA-binding activity, suggests a possible function *in vivo*. Gel filtration analysis of cell lysates show that RodZ eluted at a broad range near the 670 kDa marker, corresponding to the superstructure. The detection of this putative superstructure in living bacteria may explain why RodZ was distributed in cells as spots rather than in a continuous line. In early studies using conventional microscopy found that fluorescently-tagged MreB and RodZ from inducible plasmids colocalize as a helix-like region [6, 7], whereas TEM observations did not detect such a structure [33]. When we used SIM imaging for immunodetection of RodZ in bacterial cells, we observed RodZ signals as scattered dots. Measurement of the areas resulted in distribution among discrete values (Fig 6D), which could relate to the assembly of RodZ hexamer for the superstructure observed in the experiments described above. This observation was also consistent with the limited number of intracellular RodZ molecules (~650 copies per cell [8]) that would not be enough to form continuous helices. In addition, the result further suggests that the filamentous structures observed using TEM were artifacts (Fig 1D).

This study achieved the first super-resolution immunodetection for RodZ and MreB. Recent observation using a functional fusion MreB-msfGFP^sw^ doesn’t detect any continuous structure, whereas it localizes in scattered patches, preferentially localizes to inwardly curved areas to make feedback system keeping the cylindrical uniformity of cells [10]. This polymer of MreB, comprises a limited number of molecules (approximately 10), that varied among the deletion constructs of RodZ alleles, suggesting that RodZ acts as an assembly factor for MreB [12]. Altered MreB distribution in the Δ*rodZ* strain (Fig 6B) [12] also support this idea.

Distribution of RodZ was also changed to dense staining in the Δ*mreB* strain [8] suggesting both proteins cooperatively affect the distribution within bacterial cell. Since our results indicate self-assembly of hexamer and superstructure formation of RodZ, the cooperative effect would place both complex to the proper position within the cell.

Our observation consistently detected the possible MreB complex in gel filtration fraction near the 158 kDa standard (Fig 4B, fractions 4 to 6). In addition, microscopic observation detected colocalization of MreB and RodZ in the wild-type strain, where most of the both proteins colocalized but some part of the larger MreB signals were separately detected as red fluorescence (Fig 6C). Such “loose interaction” was also supported in gel filtration where limited MreB was co-eluted with the large complex of RodZ (Fig 4B, fractions 1 and 2). The loose interaction between RodZ and MreB might play an important role that enables flexible change for rapid growing cell body.

Our result provided the first evidence for formation of superstructure by homohexamers of RodZ. Intracellular RodZ was detected as dot-like clusters colocalizing with MreB, consistent with the superstructure formation essential for the RNA-binding activity *in vitro*.

## Supporting information

S1 Fig Purified RodZ deletion proteins. CBB-stained SDS-PAGE (5–25%) gel of the purified RodZ deletion proteins.
(TIF)

S2 Fig MALDI-TOF MS analysis of the high molecular mass complex. A, Analysis without crosslinker of purified RodZ_wt_. B, Analysis after crosslinking of purified RodZ_wt_. C, Analysis without crosslinker of purified N-terminal deletion RodZ_50-337_. D, Analysis after crosslinking of purified N-terminal deletion RodZ_50-337_. Results are shown for 0–300 kDa in Fig 3.
(TIF)

S3 Fig Stabilities of *rodZ* mutant proteins with Gly-Ser substitutions. Immunoblot analysis for RodZ in Δ*rodZ* strains carrying the indicated plasmids. Strains were grown in 5 ml of LB medium containing 25 μM arabinose and kanamycin and incubated at 30°C with shaking 150 rpm for 2.5 hrs to an OD_600_ = 0.4. Rifampicin was added at time 0. Aliquots of whole cultures were mixed with 4× SDS loading buffer at the indicated times. Each sample (2 μl) was subjected to 12.5% SDS PAGE, and corresponding areas of the gels were transferred onto a single membrane and subjected to immunoblotting using the anti-RodZ monoclonal antibody 5–17 and an anti H-NS antibody (17). Experiments were performed at least three times with similar results. Representative data are shown.
(TIF)

S4 Fig Immunoblot analysis of RodZ. Strain harboring the pBAD-rodZ_wt_ was grown in 5 ml of LB medium containing 12.5 or 25 μg/ml arabinose and kanamycin, incubate at 30°C with shaking (150 rpm) for 2.5 hrs to OD_600_ = 0.4. Each sample (10 μl) was loaded onto 10% SDS PAGE, blotted and probed with monoclonal antibody 5–17. Lanes: 1, wild-type strain (MS390); 2, Δ*rodZ* strain (MS5204); 3 and 4, Δ*rodZ* strain carrying pBAD-rodZ_wt_ (MS5215).
(TIF)

S5 Fig (TIF)

S1 Raw Image (PDF)

S1 File Report for MALDI-TOF MS analysis. (PDF)
