# Supplementary figures and images for: Superstructure formation by RodZ hexamers of Shigella sonnei maintains the rod shape of bacilli
Source: PLoS One. 2020 Feb 13;15(2):e0228052. doi: 10.1371/journal.pone.0228052 (PMC7018016; doi:10.1371/journal.pone.0228052)

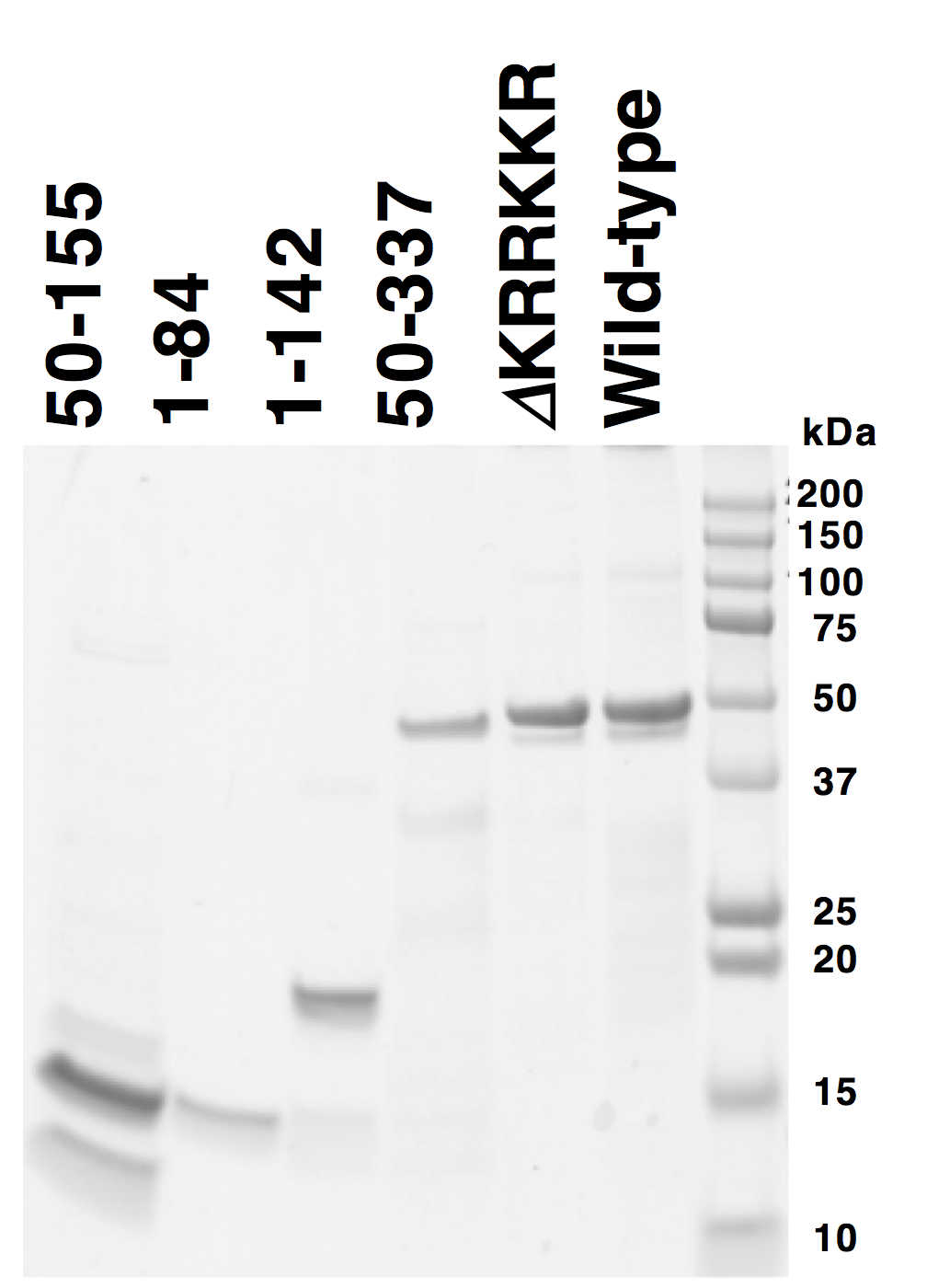

Supplement: S1 Fig — CBB-stained SDS-PAGE (5–25%) gel of the purified RodZ deletion proteins. (TIF) [file pone.0228052.s001.tif]

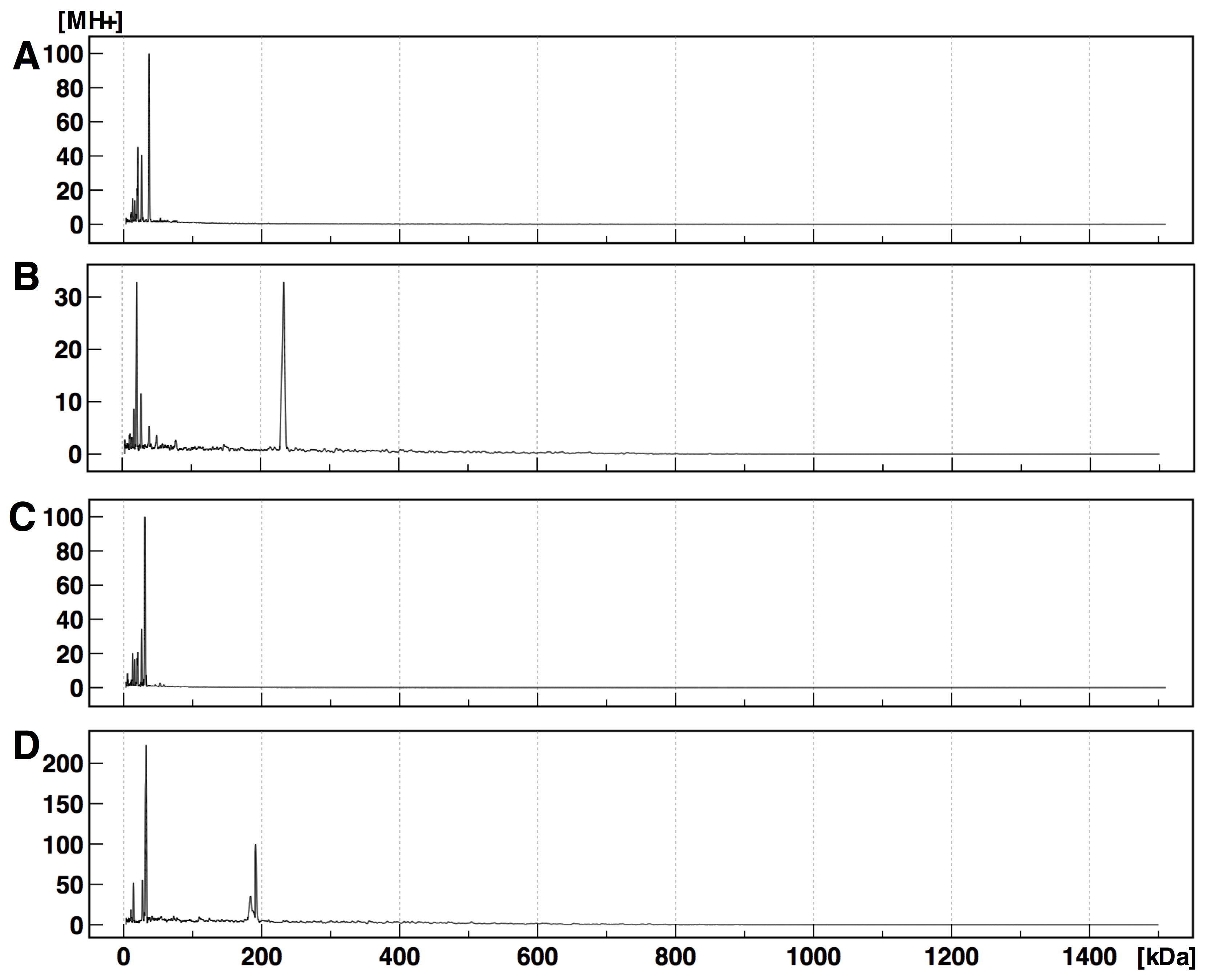

Supplement: S2 Fig — A, Analysis without crosslinker of purified RodZwt. B, Analysis after crosslinking of purified RodZwt. C, Analysis without crosslinker of purified N-terminal deletion RodZ50-337. D, Analysis after crosslinking of purified N-terminal deletion RodZ50-337. Results are shown for 0–300 kDa in Fig 3. (TIF) [file pone.0228052.s002.tif]

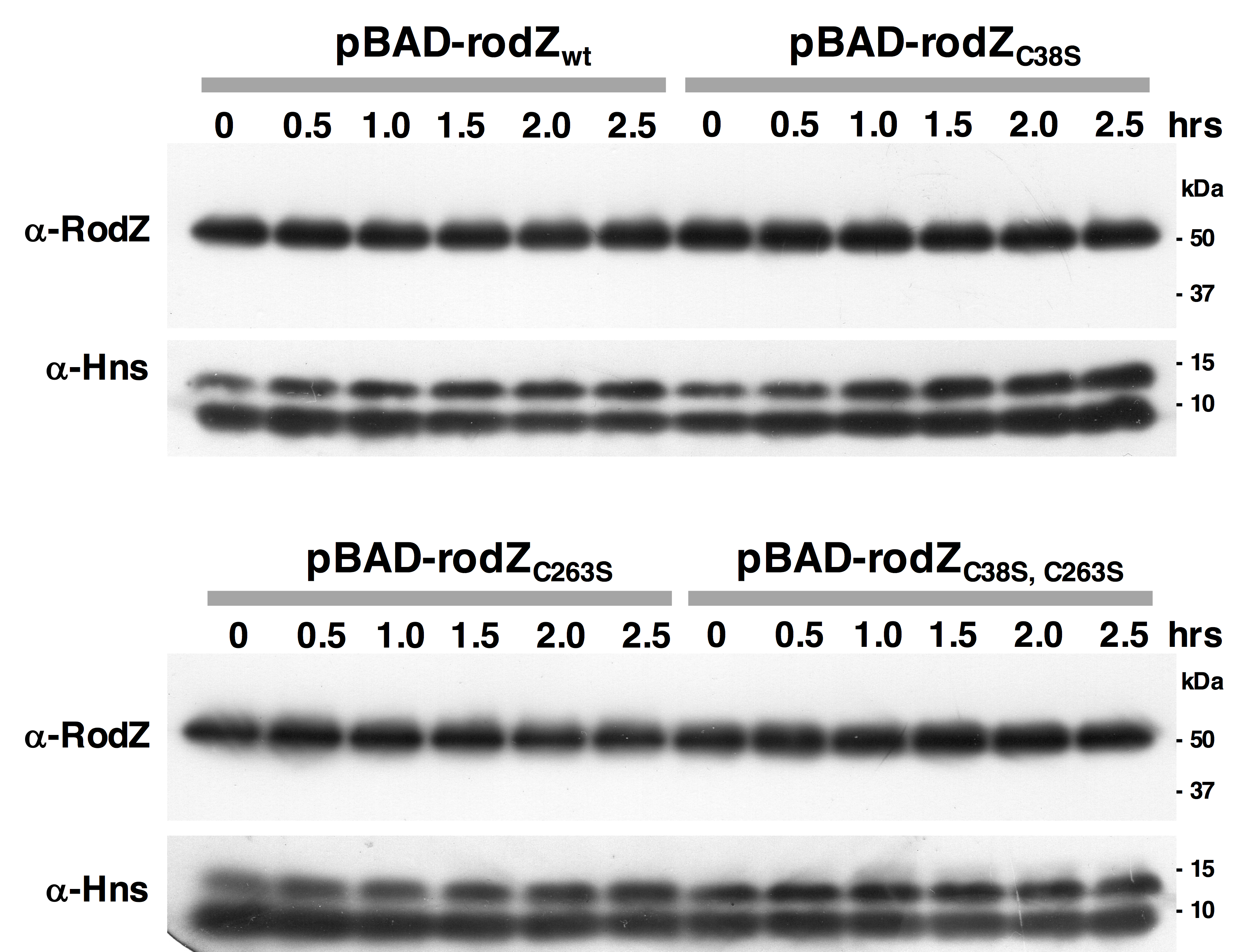

Supplement: S3 Fig — Immunoblot analysis for RodZ in ΔrodZ strains carrying the indicated plasmids. Strains were grown in 5 ml of LB medium containing 25 μM arabinose and kanamycin and incubated at 30°C with shaking 150 rpm for 2.5 hrs to an OD600 = 0.4. Rifampicin was added at time 0. Aliquots of whole cultures were mixed with 4× SDS loading buffer at the indicated times. Each sample (2 μl) was subjected to 12.5% SDS PAGE, and corresponding areas of the gels were transferred onto a single membrane and subjected to immunoblotting using the anti-RodZ monoclonal antibody 5–17 and an anti H-NS antibody (17). Experiments were performed at least three times with similar results. Representative data are shown. (TIF) [file pone.0228052.s003.tif]

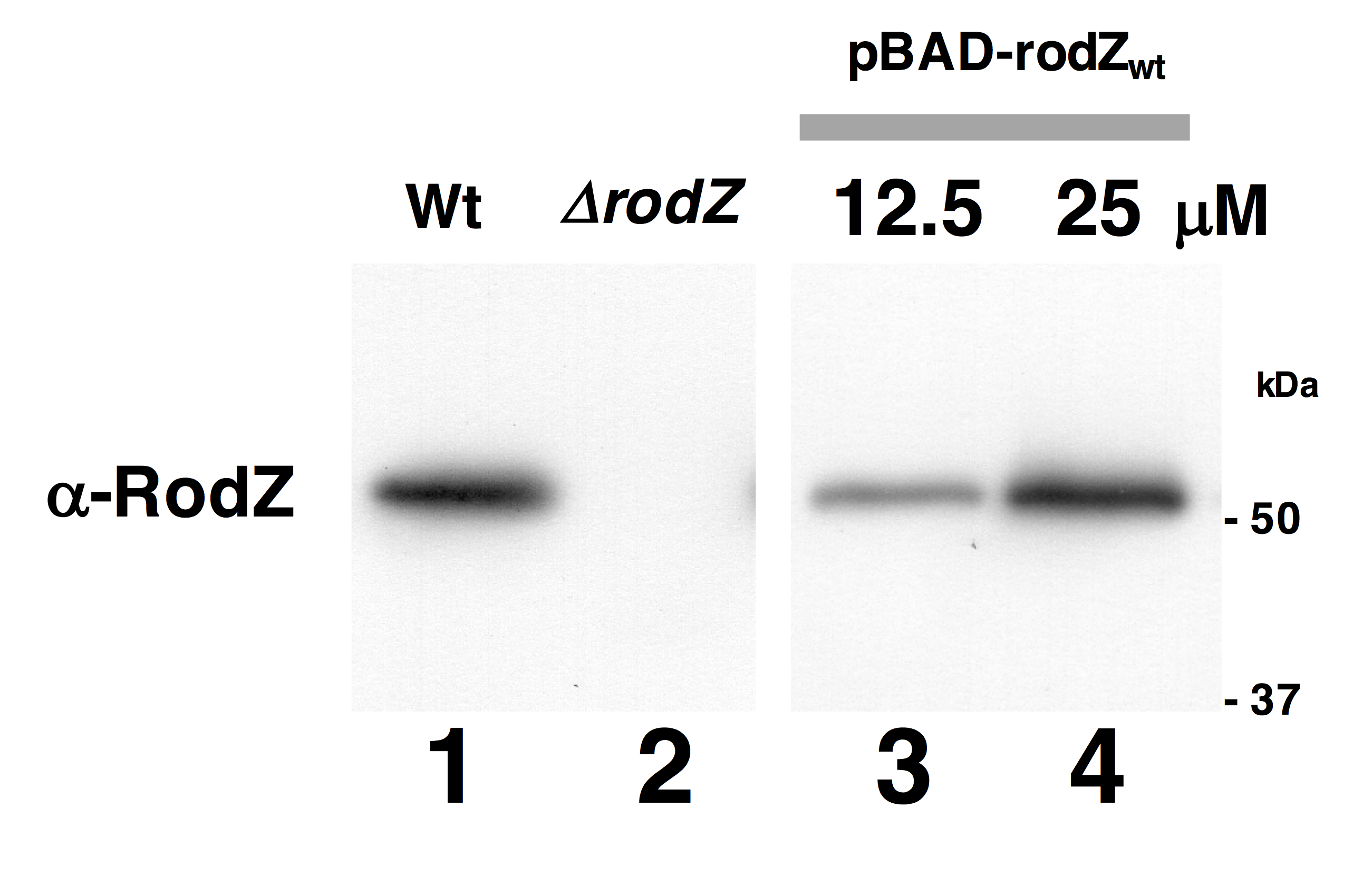

Supplement: S4 Fig — Strain harboring the pBAD-rodZwt was grown in 5 ml of LB medium containing 12.5 or 25 μg/ml arabinose and kanamycin, incubate at 30°C with shaking (150 rpm) for 2.5 hrs to OD600 = 0.4. Each sample (10 μl) was loaded onto 10% SDS PAGE, blotted and probed with monoclonal antibody 5–17. Lanes: 1, wild-type strain (MS390); 2, ΔrodZ strain (MS5204); 3 and 4, ΔrodZ strain carrying pBAD-rodZwt (MS5215). (TIF) [file pone.0228052.s004.tif]

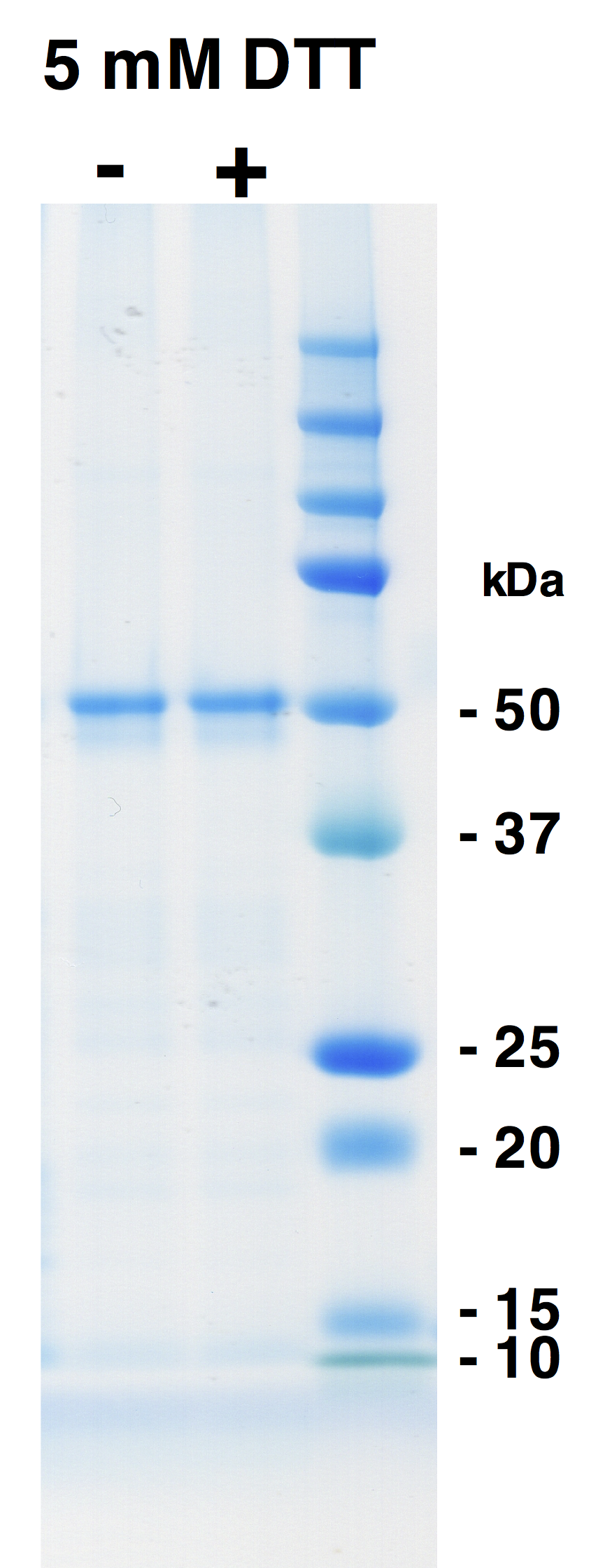

Supplement: S5 Fig — (TIF) [file pone.0228052.s005.tif]
