# Supplementary material for: Superstructure formation by RodZ hexamers of Shigella sonnei maintains the rod shape of bacilli
Source: PLoS One. 2020 Feb 13;15(2):e0228052. doi: 10.1371/journal.pone.0228052 (PMC7018016; doi:10.1371/journal.pone.0228052)

Fig. 2B

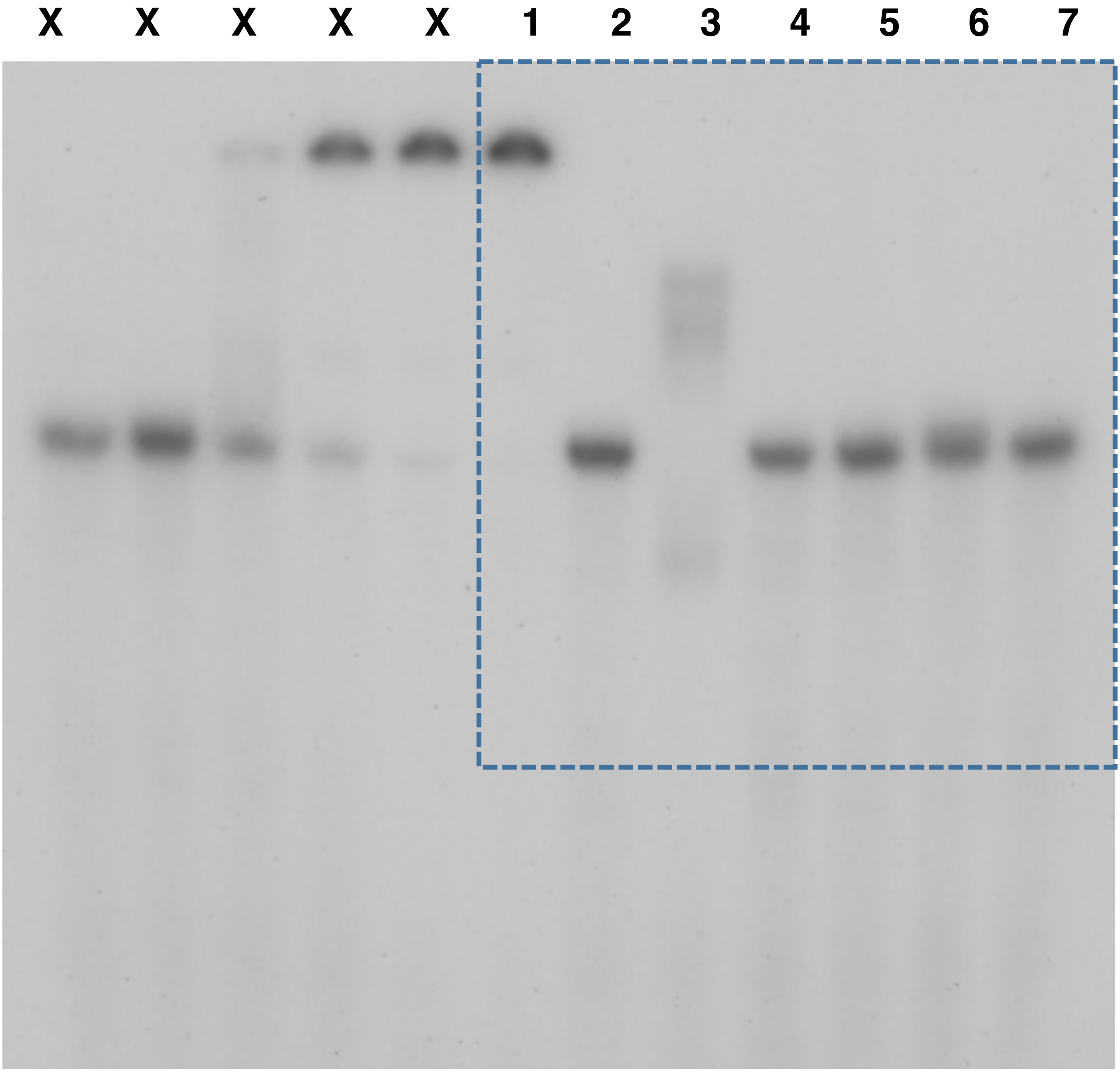

Fig. 2D

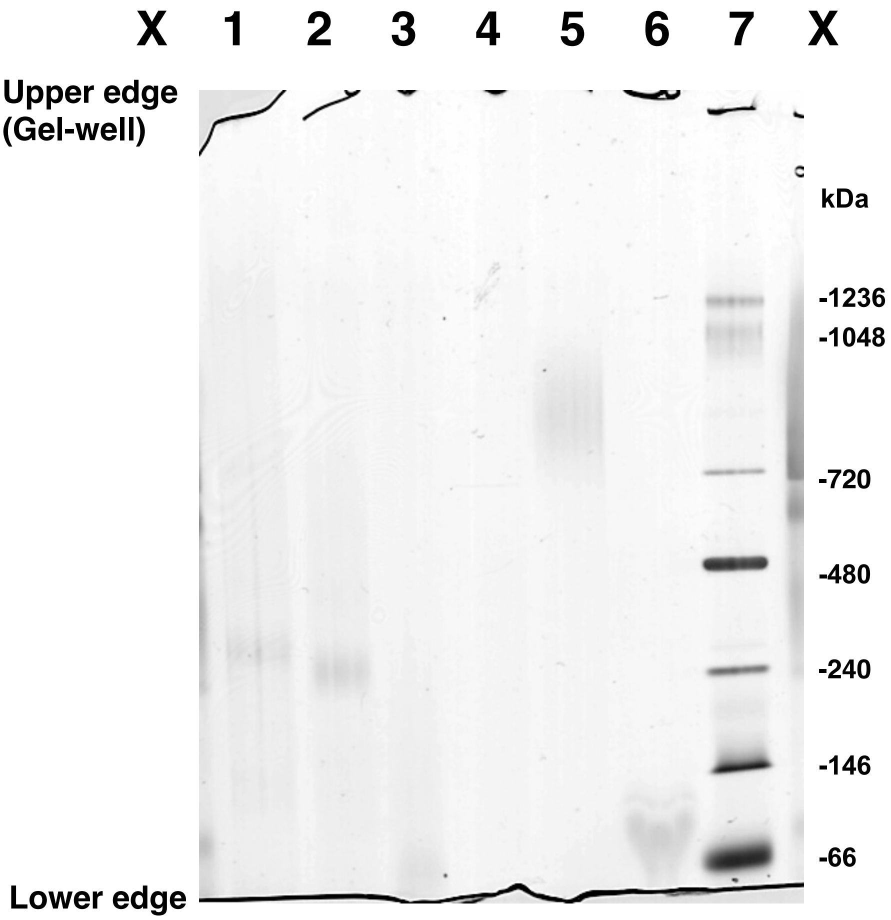

X 1 2 3 4 5 6 7 X
Upper edge
(Gel-well)
kDa
-1236
-1048
-720
-480
-240
-146
-66
Lower edge

Fig. 4A

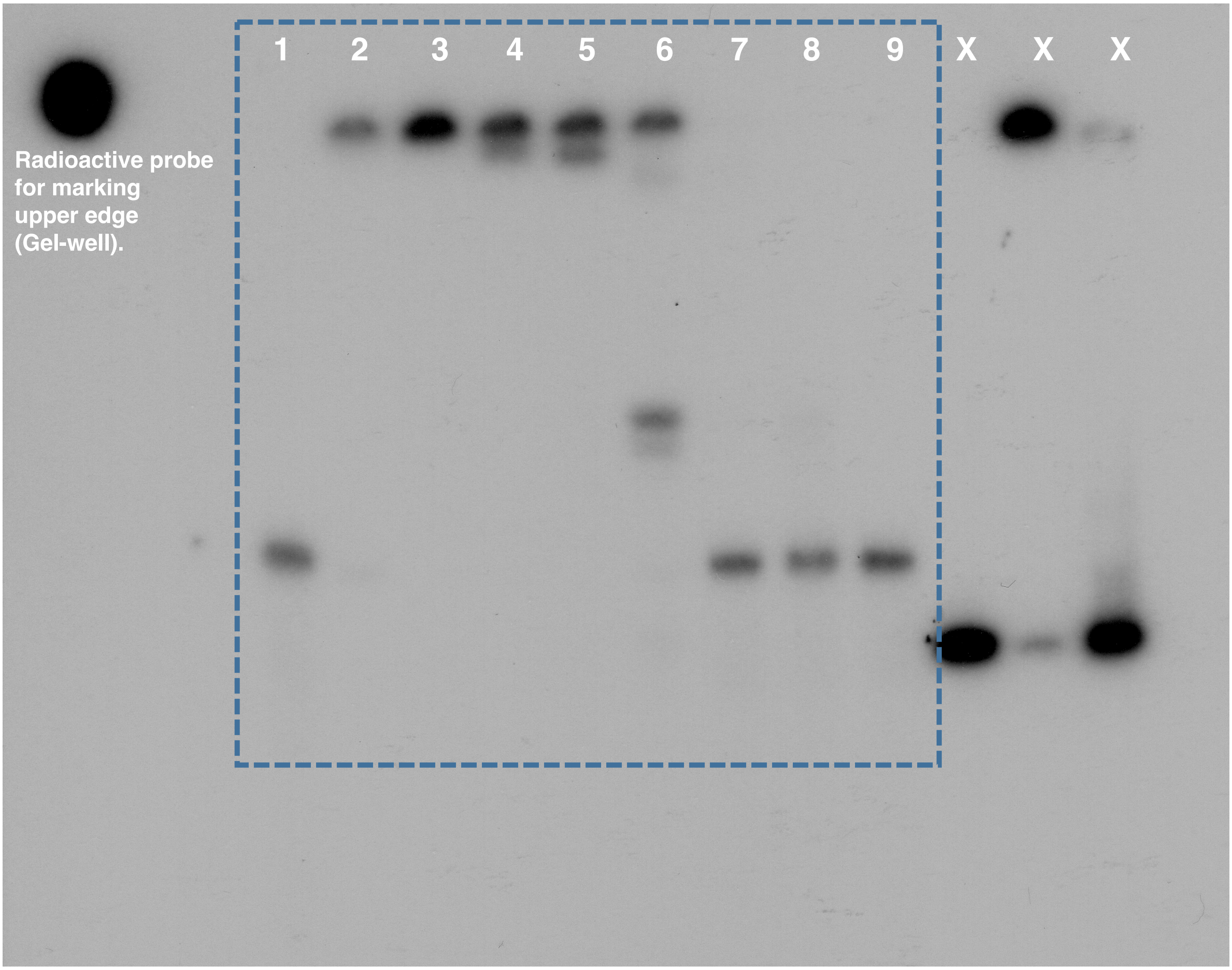
1
2
3
4
5
6
7
8
9
X
X
X
Radioactive probe for marking upper edge (Gel-well).

Fig. 4B

α-RodZ

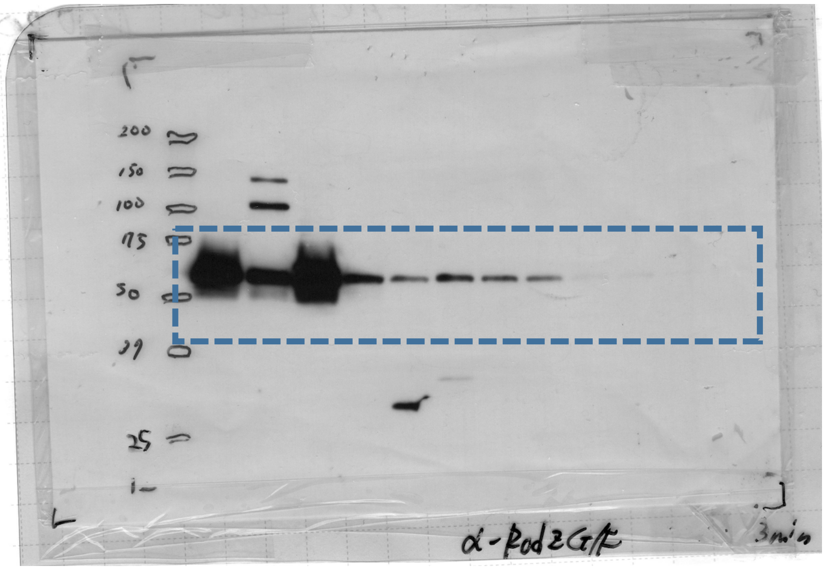

α-MreB

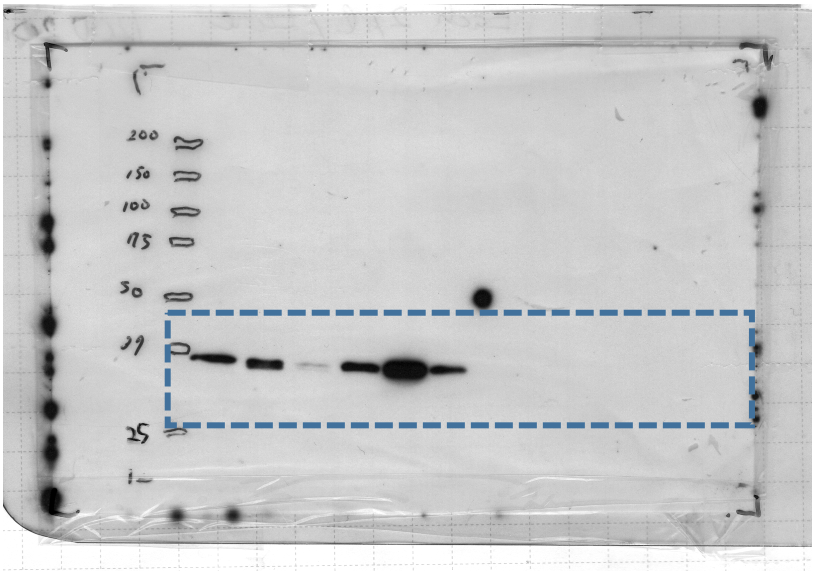

S1 Fig

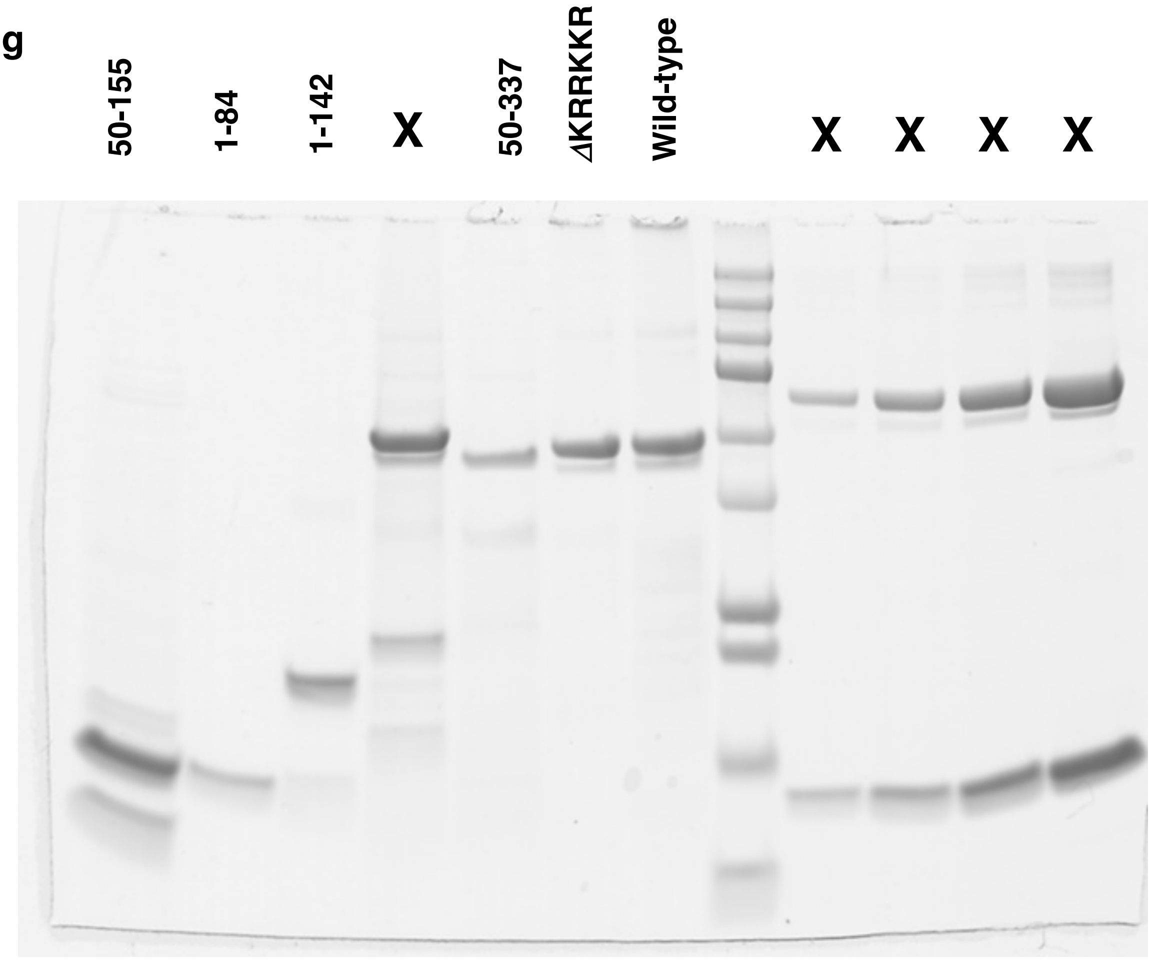

S3 Fig

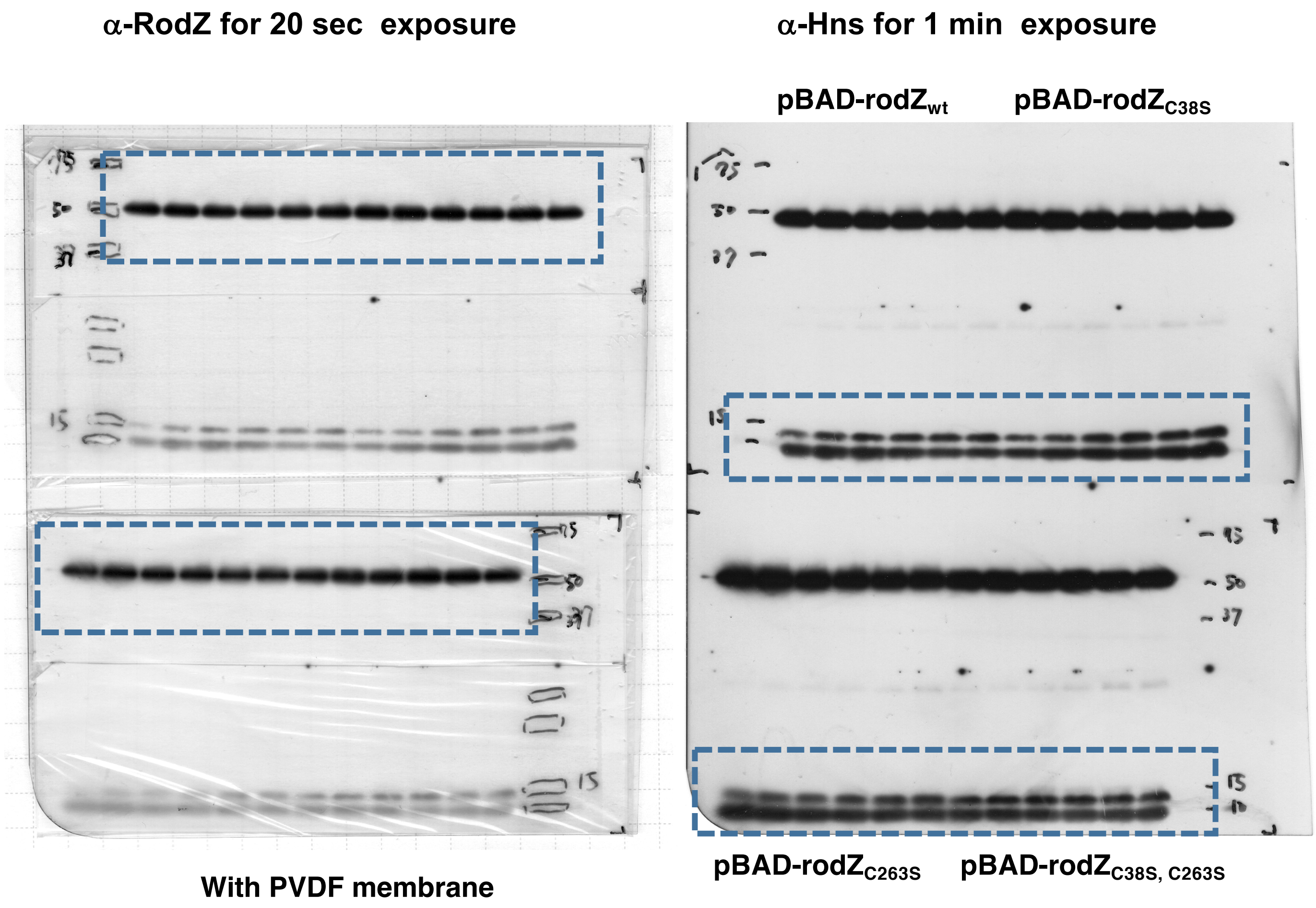

α-RodZ for 20 sec exposure
α-Hns for 1 min exposure
pBAD-rodZwt
pBAD-rodZC38S
75
50
37
15
pBAD-rodZC263S
pBAD-rodZC38S, C263S
With PVDF membrane

S4 Fig

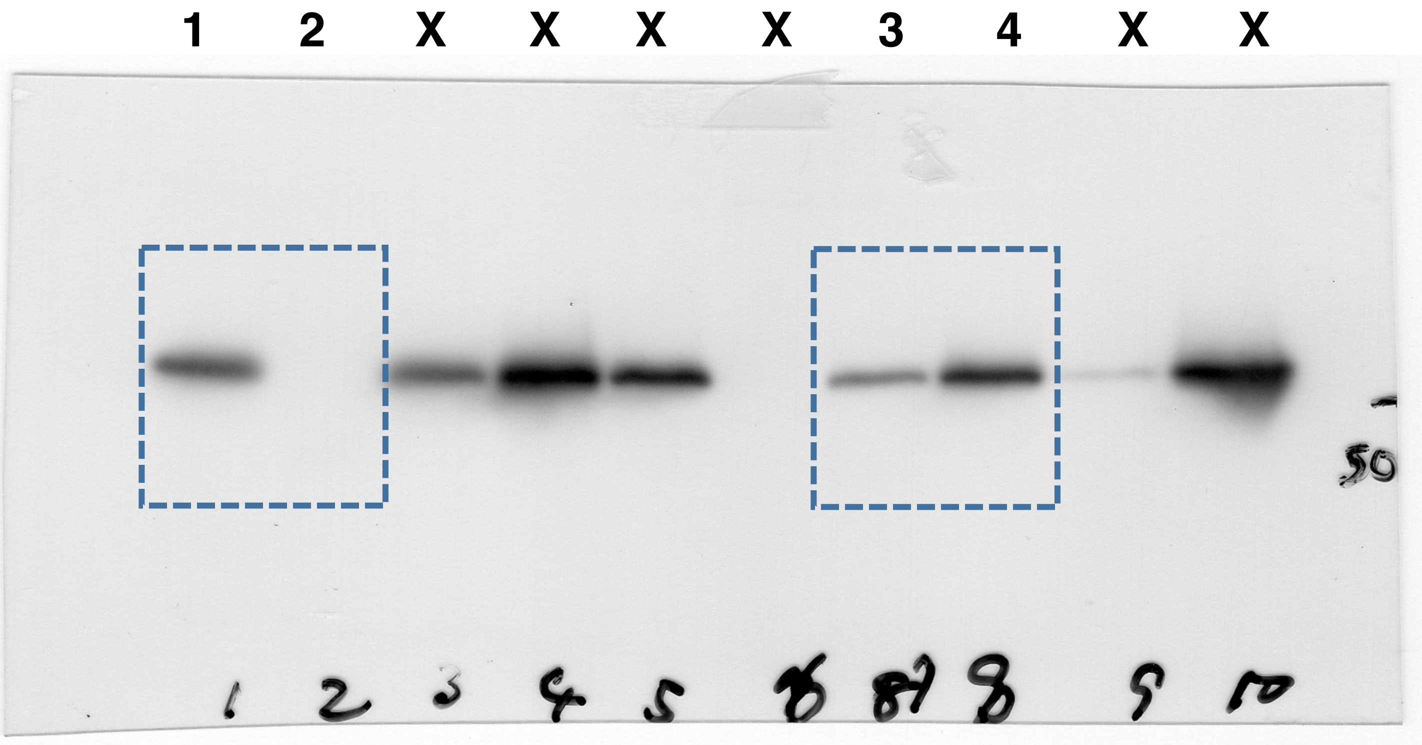

Supplement: S1 Raw Image — (PDF) [file pone.0228052.s006.pdf]
