# Supplementary material for: Superstructure formation by RodZ hexamers of Shigella sonnei maintains the rod shape of bacilli
Source: PLoS One. 2020 Feb 13;15(2):e0228052. doi: 10.1371/journal.pone.0228052 (PMC7018016; doi:10.1371/journal.pone.0228052)

Report Protein Complex Analysis
#19122012_JP_A

Samples:

- Wild- type RodZ Protein (1-337aa) (200 µL 1mg/mL Buffer : PBS)
- N-Terminal deletion RodZ Protein (50-337aa) (200 µL 1mg/mL Buffer : PBS)

<u>Characterization of molecular weight and stoechiometry characterization</u>

1. Material, Methods

1.1. Instrumentation

Mass Spectrometric Measurements (MALDI)

All the samples measurements were performed using a Bruker Ultraflex III MALDI ToF ToF mass spectrometer equipped with CovalX's HM3 interaction module. CovalX's interaction module contains a special detecting system designed to optimize protein interaction detection up to 2 MDa with nano-molar sensitivity.

1.2. Sample preparation:

Control Experiments

20µl of each protein sample were pipetted to prepare 10 dilutions of the samples with final volume 10µl. These 10 dilutions of the samples were prepared in order to obtain the following expected concentrations:

	Wild Type RodZ Protein (1-337aa)		N terminal deletion RodZ protein (50-337aa)	
Dilution	Volume	Conc.	Volume	Conc.
1	10 µl	1000µg /ml	10 µl	1000µg /ml
1/2	10 µl	500µg/ml	10 µl	500µg/ml
1/4	10 µl	250µg/ml	10 µl	250µg/ml
1/8	10 µl	125µg/ml	10 µl	125µg/ml
1/16	10 µl	62.5 µg/ml	10 µl	62.5 µg/ml
1/32	10 µl	31.2µg/ml	10 µl	31.2µg/ml
1/64	10 µl	15.6µg /ml	10 µl	15.6µg /ml
1/128	10 µl	7.8µg/ml	10 µl	7.8µg/ml
1/256	10 µl	3.9 µg/ml	10 µl	3.9 µg/ml
1/512	10 µl	1.9µg/ml	10 µl	1.9µg/ml

1 µl of each dilution obtained is mixed with 1 µl of a matrix composed of a re-crystallized sinapinic acid matrix (10 mg/ml) in acetonitrile/water (1:1, v/v), TFA 0.1% (K200 MALDI Kit). After mixing, 1 µl of each sample was spotted on the MALDI plate (SCOUT 384). After crystallization at room temperature, the plate was introduced in the MALDI mass spectrometer and analyzed immediately. The analysis has been repeated in triplicate.

Cross-link Experiments

The cross-linking experiments allow the direct analysis of non-covalent interaction by High-Mass MALDI mass. By mixing a protein sample containing non covalent interaction with a specially developed cross-linking mixture (Bich, C et al. *Anal. Chem.*, 2010, 82 (1), pp 172–179), it is possible to specifically detect non covalent complex with high-sensitivity. The covalent binding generated allows the interacting species to survive the sample preparation process and the MALDI ionization. A special High-Mass detection system allows characterizing the interaction in the High-Mass range.

Each mixture prepared for the control experiment (9 µl left) was submitted to cross- linking using CovalX's K200 MALDI MS analysis kit. 9 µl of the mixtures (from 1 to 1/32) is mixed with 1 µl of K200 Stabilizer reagent (2 mg/ml) and incubated at room temperature. After the incubation time (from 1h to 6h) the samples were prepared for MALDI analysis as for Control experiments. The samples are analyzed by High-Mass MALDI analysis immediately after crystallization.

1.3. High-Mass MALDI MS analysis

The MALDI ToF MS analysis has been performed using CovalX's HM3 interaction module with a standard nitrogen laser and focusing on different mass ranges from 0 to 1500 kDa.

For the analysis, the following parameters have been applied:

Mass Spectrometer:
Linear and Positive mode
Ion Source 1: 20 kV
Ion Source 2: 17 kV
Lens: 12 kV
Pulse Ion Extraction: 150 ns

HM3:

Gain Voltage: 3.14 kV
Acceleration Voltage: 20 kV

To calibrate the instrument, an external calibration with clusters of BSA and IgG has been applied. For each sample, 3 spots were analyzed (300 laser shots per spots). The presented spectrum corresponds to the sum of 300 laser shots. The MS data were analyzed using CovalX's Complex Tracker analysis software version 2.0.

2. Results

2.1. WildTypRODz1-337

Control Experiments

For these experiments, we detected two major peaks with MH+=26.158 kDa and MH+=37.342 kDa. These two peaks have been detected for every dilution from 1 to 1/512 (Figure 1, Control, p5).

Observed Molecular Weight (Da)	Proposed Stoechiometry
26.158	A
36.712	Wild Typ Rodz 1-337

Cross-Link Experiments

For these experiments, we detected one major non covalent complex with MH+=233.351 kDa (Figure 1, Cross-Link, p5). Using Complex Tracker Software we overlay the control and cross-link spectra. The spectrum obtained allowed to characterize the non covalent protein complex:

Observed Molecular Weight (Da)	Proposed Stoechiometry
220.455	6WildTypRodz1-337

This non covalent protein complex corresponds to an hexamer of WildTypRodz 1-337.

The spectra corresponding to the analysis are presented in Figure 1 p5.

2.2. N-terminal deletion RODz50-337

Control Experiments

For these experiments, we detected two major peaks with MH+=26.184 kDa and MH+=30.692 kDa. These two peaks have been detected for every dilution from 1 to 1/512 (Figure 2, Control, p6).

Observed Molecular Weight (Da)	Proposed Stoechiometry
26.184	A
30.692	N-terminal deletion RODz50-337

Cross-Link Experiments

For these experiments, we detected one major non covalent complex with MH+=191.059 kDa. An additional peak, related with the non covalent complex is also detected with MH+=185.798 kDa (Figure 2, Cross-Link, p6). Using Complex Tracker Software we overlay the control and cross-link spectra. The spectrum obtained allowed to characterize the non covalent protein complex:

Observed Molecular Weight (Da)	Proposed Stoechiometry
185.189	6N-terminal deletion RODz50-337

This non covalent protein complex corresponds to an hexamer of N-terminal deletion RODz50-337. The non covalent protein complex detected with MH+=180.028 may corresponds to an hexamer of 6N-terminal deletion RODz50-337 with a deletion part.

The spectra corresponding to the analysis are presented in Figure 2 p6.

Figure 1: WildtypRodZ(1-337)
[Dilution 1/4=250µg/mL
Total Volume: 10 µl
Cross-link: DSS, 180 minutes incubation time

HM3 High Mass MALDI ToF Analysis
CovalX for AMR 17/12/12

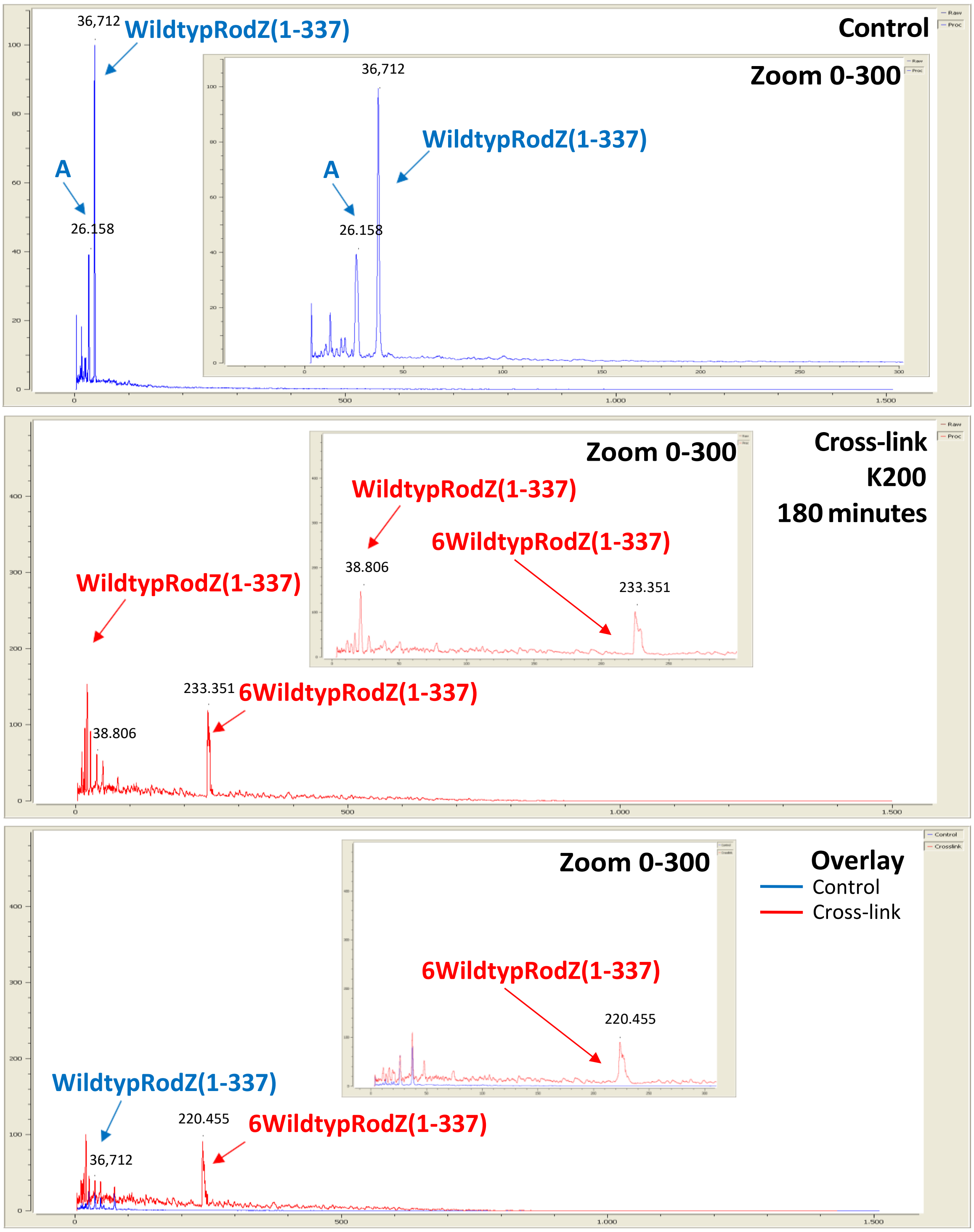

Figure2:N-TerminaldeletionRodZprotein(50-337aa) HM3 HighMass MALDI ToF Analysis

CovalX for AMR 17/12/12

Dilution 1/4=250µg/mL
Total Volume: 10 µl
Cross-link: DSS, 180 minutes incubation time

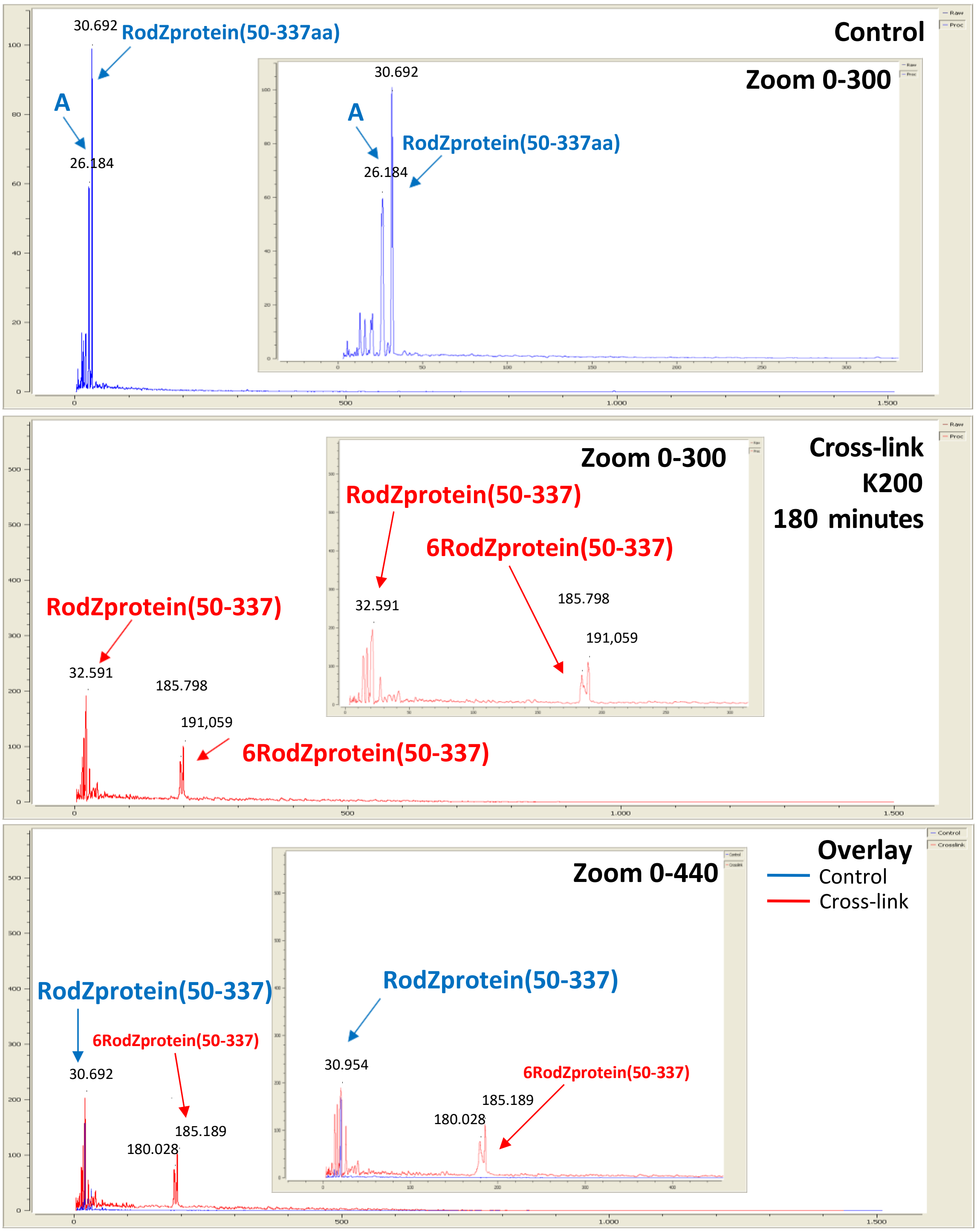

Supplement: S1 File — (PDF) [file pone.0228052.s007.pdf]
